# NLRP3 inflammasome in cardiovascular diseases: an update

**DOI:** 10.3389/fimmu.2025.1550226

**Published:** 2025-02-26

**Authors:** Binhai Mo, Yudi Ding, Qingwei Ji

**Affiliations:** ^1^ People’s Hospital of Guangxi Zhuang Autonomous Region, Nanning, China; ^2^ First People’s Hospital of Nanning, Nanning, Guangxi, China

**Keywords:** NLRP3, cardiovacsular diseases, inflammation, pyroptosis, inflammasome

## Abstract

Cardiovascular disease (CVD) continues to be the leading cause of mortality worldwide. The nucleotide oligomerization domain-, leucine-rich repeat-, and pyrin domain-containing protein 3 (NLRP3) inflammasome is involved in numerous types of CVD. As part of innate immunity, the NLRP3 inflammasome plays a vital role, requiring priming and activation signals to trigger inflammation. The NLRP3 inflammasome leads both to the release of IL-1 family cytokines and to a distinct form of programmed cell death called pyroptosis. Inflammation related to CVD has been extensively investigated in relation to the NLRP3 inflammasome. In this review, we describe the pathways triggering NLRP3 priming and activation and discuss its pathogenic effects on CVD. This study also provides an overview of potential therapeutic approaches targeting the NLRP3 inflammasome.

## Introduction

Cardiovascular disease (CVD) is a leading threat to public health in the modern era (1). Since studies have demonstrated the role of immunology in the progression of atherosclerosis (2), an increasing number of CVDs have been linked to inflammation. Despite strong evidence that inflammatory processes contribute significantly to the development and progression of CVD, the specific mechanisms underlying these processes remain unclear.

As an important indicator of damage to cardiovascular tissue and a member of the nucleotide-binding oligomerization domain (NOD)-like receptor (NLR) family, NOD-like receptor protein 3 (NLRP3) is a pattern recognition receptor (PRR). Numerous immune stimuli, such as microbial pathogen-derived molecules and extracellular or intracellular damage-associated molecular patterns, can activate NLRP3. NLRP3 recruits apoptosis-associated speck-like protein containing a caspase recruitment domain (ASC), which is linked to procaspase-1, to assemble the inflammasome. It promotes the maturation of IL-1β and IL-18. Both innate and adaptive responses are involved in the inflammatory mechanisms of CVD. IL-1β and IL-18 are key mediators of these mechanisms, and preclinical and clinical data from studies of drugs that target these molecules are available (3, 4). The role of the NLRP3 inflammasome in CVD is outlined in this review, along with the latest advances and potential therapeutic benefits of blocking the NLRP3 inflammasome.

## NLRP3 inflammasome

Since its initial discovery, inflammasome studies have emerged as the main focus of innate immunity research. Leukocytes express NLRP3, as do other cardiovascular system cells, such as cardiomyocytes (5). Direct induction of inflammasome formation is a crucial function of intracellular PRRs. NLRP1, NLRP3, NOD-, LRR-, pyrin, absent in melanoma 2 (AIM2) and caspase recruitment domain (CARD)-containing protein 4 (NLRC4) are the five intracellular PRRs that are known to be inflammasome receptors (6). NLRP1, NLRP3, NLRC4, Pyrin, and AIM2 each play distinct roles and causes. NLRP3 is activated by numerous pathogen-associated molecular patterns (PAMPs) and damage-associated molecular patterns (DAMPs), and it separates from other proteins (7). The three components of the NLRP3 inflammasome include procaspase-1, apoptosis-associated speck-like protein containing a CARD (ASC), and NLRP3 (8). The NLR proteins have a bilateral organization: the central domain NODS (9), the C-terminal LRR domain, and the N-terminal effector domain (10, 11). NLRP3 uses pyrin as the N-terminal effector and shares the same structure as NLR.

ASC is a protein that resembles a speck and connects procaspase-1 to NLRP3. An N-terminal PYD and a C-terminal CARD make up its two domains (12). Through the homotypic CARD–CARD reaction, the CARD on ASC could be linked to procaspase-1, and the PYD on ASC establishes a strong bond with pyrin on NLRP3 via a comparable connection (13–15). Procaspase-1 oligomerization on ASC fibers facilitates proximity-driven autocatalytic caspase-1 evolution in addition to electing procaspase-1 and establishing a connection with PYD on NLRP3 (16). The effector protein of the inflammasome is called procaspase-1. Autocatalytic evolution involves the breakdown of procaspase-1 into caspase-1. For human homeostasis, cleaved caspase-1 is an essential proteolytic enzyme. An active heterotetramer formed by caspase-1 cleaves pro-IL-1β and pro-IL-18 into cultured cytokines and triggers their release (17). In macrophages, the assembly of inflammasomes is arranged later. The membranes of innate immune cells highly express toll-like receptors (TLRs). When TLRs are triggered by PAMPs or DAMPs, blocked nuclear factor-κB (NF-κB) is phosphorylated and lysed, releasing active NF-κB. After that, NF-κB meets the nucleus and promotes the transcription of NLRP3, pro-IL-1β, and pro-IL-18 (18). Tumor necrosis factor receptor (TNFR) and IL-1β receptor (IL-1R) can work similarly in priming NLRP3 via the authorized TLR–NF-κB pathway. The NLRP3 inflammasome product, IL-1β, can also aid in priming NLRP3 itself because TNFR and IL-1R can trigger the NF-κB pathway, which orderly induces NLRP3 transcription (19).

New research has revealed circadian fluctuations in the process of NLRP3 expression. In addition to key NF-κBs, several nuclear receptors play crucial roles in rhythmic pattern regulation, which was initially observed in mouse and human primary macrophages (20). The circadian clock timing of the NLRP3 inflammasome is dependent on mitochondrial function and driven through the circadian gene Bmal1 (21). Melatonin is a ubiquitous hormone with a circadian rhythm, and studies have shown that melatonin alleviates NLRP3 inflammasome activity (22).Rev-erbα and retinoic acid receptor-related orphan receptor γ (RORγ) are members of the nuclear receptor family. These two receptors connect to equal promoter locations, regulating NLRP3 priming (23). Furthermore, Rev-erbα has adverse effects on priming and triggering of the NLRP3 inflammasome and is circumstantial in the NF-κB pathway (24, 25). Human pathology studies have shown evidence of NLRP3 inflammasome activation in CVD (26). Patients with AMI also have elevated plasma levels of NLRP3 and caspase 1 (27). The time-dependent activation of the NLRP3 inflammasome in the heart has been examined in animal studies (28). In a mouse model of AMI, the expression of NLRP3 inflammasome components increased over time, whereby activation occurred 3–24 h after reperfusion, peaking after 1 or 3 days in mice with reperfused and nonreperfused AMI, respectively (29). The notion that NLRP3 expression is regulated in a time-dependent manner is new, but it has been confirmed and will expedite further investigations into the development of cardiovascular and inflammatory diseases.

Pyroptosis is one of the specific effects of the NLRP3 trigger. Pyroptosis is a unique type of programmed cell death (30, 31). The inflammasome acts as a mediator of pyroptosis. The specific mechanism is as follows: Gasdermin D (GSDMD), a unique protein, is split into two fragments by caspase-1 on the NLRP3 inflammasome. The N-terminus forms membrane pores, leaking IL-1β and IL-18 and triggering pyroptosis (32).

## Activation of the NLRP3 inflammasome

**Figure 1:**
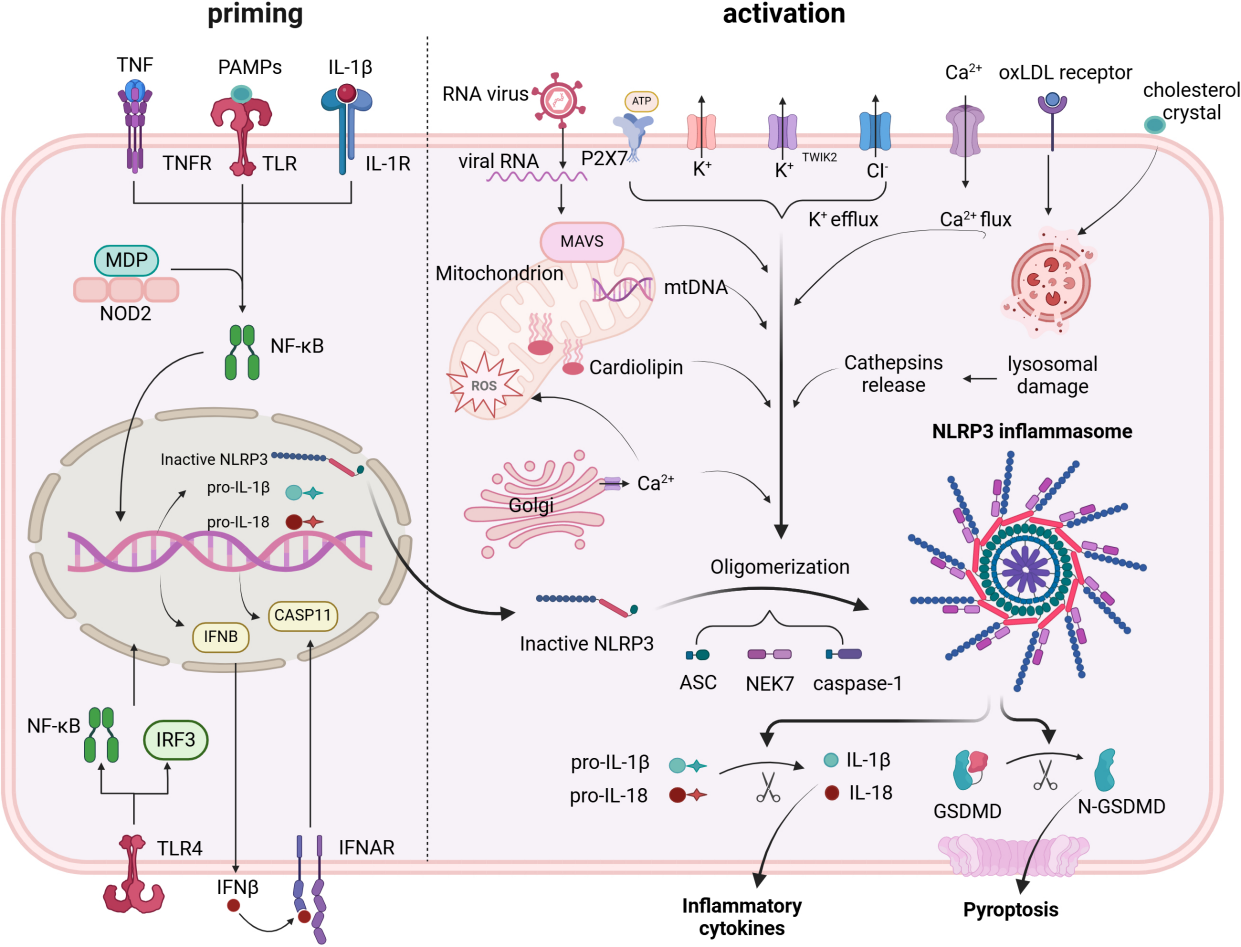
Priming and triggering signals control the formation and activation of the NLRP3 inflammasome. Priming signals lead to the transcription of NLRP3 inflammasome components, mainly via the NF-κB pathway. Triggering signals cause: assembly of the NLRP3 inflammasome; activation of caspase-1; conversion of pro-IL-1β and pro-IL-18 into their mature forms; and cleavage of GSDMD, which creates pores in the cell membrane, facilitating the release of active IL-1β and IL-18.

Two separate stimuli are needed to activate the NLRP3 inflammasome, which is tightly controlled at different levels. Primarily PAMPs and DAMPs, the initial signals activate PRRs or other receptors and trigger the reproduction of NLRP3, pro-IL-1β, and pro-IL-18 through the NF-κB signaling pathway. NLRP3 is activated and its inflammasome assembly is encouraged by the second signal. In general, stimulus events fall into three primary categories: damage to or malfunction of cell organelles, reactive oxygen species (ROS) and ionic flux (33) (**Figure 1**).

### Ionic flux

Prior to the identification of the inflammasome, it was demonstrated that cytosolic K+ depletion facilitated the evolution and clemency of IL-1β (34). The NLRP3 inflammasome trigger was inhibited by increasing K+ efflux with increasing extracellular potassium concentration, whereas the inflammasome trigger was provoked by a decrease in the intracellular K+ concentration. The purinergic receptor (P2X7R) facilitates the triggering of the NLRP3 inflammasome, which is frequently activated by ATP (35). K+ efflux occurs in conjunction with P2X7R engagement. In addition to the traditional activating pathway, the NLRP3 inflammasome is triggered by the efflux of K+ through noncanonical pathways (36, 37).

Numerous intercellular signaling pathways are closely linked to the activation process and involve the mobilization of calcium. Using BAPTA-AM to chelate Ca2+ was demonstrated to suppress IL-1β flow in early studies, implying that Ca2+ signaling may be involved in the triggering the NLRP3 inflammasome (38). Different stimuli, such as extracellular ATP, nigericin and particulates, can induce Ca2+ mobilization from the endoplasmic reticulum (ER) or the extracellular space, leading to mitochondrial damage and subsequent NLRP3 activation (39). One of the sensors of extracellular Ca2+ is the calcium-sensing receptor (CaSR), which seems to mediate the increase in intracellular Ca2+ and the decrease in cellular cyclic AMP (cAMP). Both events are associated with NLRP3 activation (40). In rats, CaSR mediates NLRP3 activation in the context of hypertension and AMI (41, 42). The trigger appeared to be limited when murine CASR was knocked out (40). However, a complete illustration of the precise mechanism is lacking. NLRP3 and ASC may interact more easily if Ca2+ facilitates conformational changes.

The activation may also be significantly influenced by other ion concentration changes. The decrease in K+ efflux from the NLRP3 trigger increased when Na+ influx was blocked by lowering the extracellular concentration, which blocked its activation. The intracellular osmotic pressure increased when crystals of monosodium urate were used to increase concentrations within the cell and prevent the influx of Na+. This resulted in water inflow and cell swelling, which lowered the relative potassium concentration and prevented its efflux and subsequent feedback, which significantly triggered NLRP3 inflammasome activation (43). Since increasing extracellular chloride concentrations promote the evolution of IL-1β triggered by ATP, chloride ions also play a key role in triggering this process (44).In addition, the NLRP3–Nek7 interaction, a downstream response of mitochondrial dysfunction that controls the trigger of the NLRP3 inflammasome, can be facilitated by chloride intracellular channels (CLICs), according to recent research (45). However, studies have revealed that CLICs contribute to the reproduction of pro-IL-1β and the development of ASC-speck, even when the level of NLRP3 is not affected (46).

### Reactive oxygen species

Although they are a wide range of substances, reactive oxygen species are primarily side products of different aerobic metabolism procedures (46, 47). ROS and the initiation of NLRP3 in treated cells were found to share similar trigger signals in the original study (46). ROS are produced by NADPH oxidase (NOX). Blocking NOX or NOX2 affects the initiation of the NLRP3 inflammasome in human or mouse cells (48–50). Additionally, NLRP3 is activated by the oxidative process of lipid acids, which is facilitated by NADPH oxidase 4 (NOX4) (51). Research has shown that ROS inhibitors hinder the signaling pathway of NLRP3 initialization, suggesting a novel perspective on ROS function (52). On the basis of all of the aforementioned data, the ROS pathway significantly influences NLRP3 activation; however, the various elements are intricate and need further investigation to determine their roles.

### Mitochondrial dysfunction

In mitochondria, the respiratory response is a series of events. The oxygen used in the respiratory chain may accumulate as mitochondrial ROS (mtROS) once the chain process is stopped. Nakahira and colleagues previously demonstrated that mtROS are necessary for the reaction of NLRP3 to ATP and LPS. In sepsis models, mice lacking mitochondrial protective proteins have increased mtROS levels and release further amounts of IL-1β and IL-18 (53). mtDNA is another byproduct of mitochondrial dysfunction. The mtDNA released from dysfunctional mitochondria was found to perform oxidation and exert a crucial influence on activation, in addition to interacting with NLRP3 (54). Subsequent research demonstrated that the activation process depended heavily on mtDNA synthesis triggered by the TLR signaling pathway (55).

### Lysosomal damage

The NLRP3 inflammasome has been shown to be activated in macrophages by particulate matter (56–58). Owing to the inability of active lysosomal enzymes to break down these particles, lysosomal disruption, acidification, and cytosolic leakage of lysosomal contents occur. The hydrogen balance between the cytosol and lysosomes is preserved by the proton pump H+ ATPase. The application of the H+ ATPase blocker bafilomycin A also prevented particulate matter-induced NLRP3 triggering, suggesting that lysosomal acidification is a significant factor in the activation process (48). The particulate matter-induced triggering of the NLRP3 inflammasome is also related to the outflow of enzymes that are active within lysosomes. Cathepsin B, a key lysosomal enzyme, is necessary for the release of IL-1β but has no effect on the synthesis of pro-IL-1β. Additional studies on cathepsins L, C, S, and X revealed similar findings. A single cathepsin’s promoting role in activation may be masked by functional redundancy because of the wide substrate specificities of cathepsins (59). Overall, the release of active lysosomal enzymes is an inevitable part of life, but other mechanisms can compensate for their lack.

## The role of the NLRP3 inflammasome in cardiovascular diseases

### Atherosclerosis

Atherosclerosis is widely recognized as the main cause of numerous CVDs (60). Passive lipid deposition, proliferation of vascular smooth muscle cells, and infiltration of leukocytes are its three key characteristics (61). Initially, the mechanism was ascribed to the effects of lipid accumulation (62). However, the role of inflammatory responses has attracted increasing attention as research has progressed. Numerous experimental and clinical investigations have suggested that numerous inflammatory cytokines are involved. Owingwell et al. were the first to explicitly highlight the role of NLRP3 in the atherogenesis process (56). They employed an LDLR-deficient (LDLR-/-) mouse model and then transplanted bone marrow from wild-type, Nlrp3-/-, Asc-/-, or IL-1α-/-/IL-1β-/-mice to establish a high-fat diet. According to these findings, mice that received transplants of Nlrp3-/-, Asc-/-, or IL-1α-/-/IL-1β-/-marrow had fewer lesions than did the wild-type mice. In 2012, Usui et al. generated Apoe-/- Casp-/- mice by mating Apoe-/- mice with Casp1-/- mice (61). They nourished mice with a Western diet and discovered that the lesions were also reduced without changing the distribution of lipoproteins and cholesterol or the total serum cholesterol levels. That year, Gage et al. fed Apoe-/- Casp-/- mice a Western diet with 1–5% cholesterol and observed comparable outcomes (61). These findings suggest that the NLRP3-induced inflammatory process plays a significant role in atherogenic lesions. These findings do not seem to be definitive, as some studies have reported conflicting findings (63–65). Nonetheless, variations in sex, feeding circumstances, and experimental model selection can account for those differences. It might be better to use an LDLr-/- mouse model to simulate human atherosclerotic disease.

Cholesterol crystals (CCs) are generally regarded as the key initiators triggering the NLRP3 inflammasome, although the precise mechanism varies among studies (65, 66). Like other crystalline NLRP3 energizers, CCs work by causing lysosomal damage to activate NLRP3 (67). Research has demonstrated that lysosomal dysfunction and IL-1β excretion are decreased when transcription factor EB expression is increased. This transcription factor induces lysosomal and autophagy genes in response to lysosomal stress (68). The development of enlarged atherosclerotic plaques was also observed in Apoe-/-mice treated with increased CCs and lacking macrophage-specific autophagy (69). These findings also demonstrate how macrophage autophagy reduces CC-mediated NLRP3 activation, which has an atheroprotective effect.

The priming of the NLRP3 activation process is another function of CCs in addition to the traditional lysosomal damage activation pathway. Complement system activation by CCs (70), which supplies the signaling pathway of priming *in vivo*, was demonstrated via whole-blood composition analysis. Neutrophil extracellular traps (NETs), which have been shown to be involved in the activative track, are also released by CCs (71). Tests conducted on mice lacking NETs revealed the absence of NET-related catalysts, including neutrophil elastase (NE) and neutrophil proteinase 3 (PR3), which have been implicated in the cleavage of pro-IL-1β. Naturally, mice lacking NETs also present fewer atherogenic lesions (72).

Another important factor in NLRP3 activation is LDL cholesterol (LDL-C) (73). Since atherogenesis is closely associated with blood cholesterol levels, particularly LDL-C, LDL-C is inherently regarded as a significant risk factor for CVD (74). Traditional conservative therapy has been verified to be successful in lowering cardiovascular event risk and focuses on regulating plasma cholesterol levels. However, not all patients respond well to this treatment (75), and clonal hematopoiesis patients might not benefit as much from ezetimibe or statin therapy. Through enzymatic or nonenzymatic processes, the body breaks down LDL to produce oxLDL, the actual cause of atherogenesis (76, 77). Scavenger receptors (SRs) of the cluster of differentiation (CD) 36 type can facilitate the internalization of oxLDL into macrophages (78). As previously stated, oxLDL causes the formation of intracellular crystalline cholesterol after absorption, which in turn triggers the assembly of the NLRP3 inflammasome through lysosomal damage (56). Furthermore, new studies have shown that oxLDL can attach to CD36 and then facilitate the formation of TLR4/6 heterodimeric complexes, which in turn primes activation (79).

Studies have shown that calcium phosphate crystals and ATP are two small molecules that are closely linked to vascular calcification as well as calcified atherosclerotic lesions, in addition to these two main proatherosclerotic substances. Like CCs, calcium phosphate crystals participate in the NLRP3 activation process, influence the caspase-1 signaling pathway, and subsequently facilitate the splitting and unleashing of IL-1α and IL-1β (61). Additionally, a key activation trigger is the extracellular ATP emitted by dead cells, and the ATP-dependent initiation pathway is mediated by the central receptor P2X7R. LDL-/- and Apoe-/- mice presented increased P2X7R expression. Consequently, small lesions were observed in LDL-/- mice when P2X7R was knocked down. Compared with control mice, Apoe-/- and P2X7R-/- mice presented comparable outcomes. These findings indicate that the ATP-dependent NRLP3 inflammasome activation pathway is activated by extracellular ATP (80, 81). One study, however, revealed that IL-1β treatment prevented positive outward remodeling but was unable to restrict the size of the lesion (82). These findings suggest that a simple summary of the detrimental role of IL-1β in atherosclerotic development is inappropriate. However, the beneficial effects of IL-1β are still debatable since it encourages plaque rupture, vascular wall stiffness and vascular calcification.

### Pericarditis

Numerous infectious and noninfectious factors can contribute to pericarditis, a common condition in the pericardium (83). Despite being typically self-limiting, the threat of developing multiple complications and relapses in the affected population has increased by more than 30% when standard therapies are used (84). Acute pericarditis can be treated with only a small number of approved medications because the pathophysiology of the condition is still poorly understood (85). Currently, colchicine is regarded as the primary therapy for both acute and recurrent pericarditis (86). It is used in anti-inflammatory treatments as a microtubule polymerization inhibitor. In recent studies, colchicine has been shown to inhibit the stimulation of the NLRP3 inflammasome, which can hinder the presentation of irritant agents and block microtubule assembly (33, 87). Furthermore, Anakinra, a recombinant IL-1 Ra, was found to reduce the potential for recurrence in patients with pericarditis who were resistant to colchicine in the Anakinra—Treatment of Recurrent Idiopathic Pericarditis (AIRTRIP) study (88). These findings suggest that the NLRP3 inflammasome/IL-1 pathway might be vital to the development of pericarditis.

To investigate the variation in inflammatory molecules, pericardial specimens from individuals with chronic pericarditis were examined prior to *in vivo* experiments (89). The results of the immunofluorescence staining revealed that, in contrast to that in the control samples, the level of ASC was elevated in the human pericardial samples. Compared with those in the controls, the expression levels of NLRP3 and caspase-1 were notably elevated in the pericarditis patients, highlighting the key elements of pericarditis development and confirming the theory of medication.

Mauro and associates were the first to validate the therapeutic impact of blocking the NLRP3 inflammasome in a mouse model of acute pericarditis (90). To create the pericarditis model, CD-1 male mice were given an injection of zymosan A, a yeast-derived NLRP3 stimulator, into their pericardial sac. To assess the degree of pericardial inflammation, three parameters were measured: pericardial thickness, ASC expression, and pericardial effusion. Compared with the sham mice, the treated mice presented greater effusion three days following surgery. Visceral pericardial thickness and ASC expression are highly consistent seven days after surgery (89). The NLRP3 inhibitors 16673-34-0, colchicine, and nonsteroidal anti-inflammatory drugs decreased pericardial effusion or pericardial thickness in subsequent recovery experiments. In model mice, pericarditis syndrome was also alleviated, and inflammasome formation was reduced by the use of anakinra and an IL-1 trap. Injury to pericardial cells induces the release of intracellular contents. Pro-IL-1α released outside the cell is already active, binds to the IL-1 receptor type I (IL-1RI) on macrophages and activates the signal, functioning as an alarmin. Macrophages respond to alarmins by forming and triggering the NLRP3 inflammasome, which processes and releases active IL-1β in its active form (91). This process amplifies the inflammatory response and induces further injury, which in turn leads to the release of more pro-IL-1α. IL-1β also induces the release of IL-6, which mediates the acute phase reaction associated with inflammation (92). Together, these data validate the role of the NLRP3 inflammasome and IL-1 in pericarditis. Acute and recurrent pericarditis may benefit from blocking the NLRP3 and IL-1 pathways, especially when coupled with the favorable therapeutic effects of colchicine and anakinra.

### Hypertension

Hypertension, or systemic arterial hypertension, is a prevalent risk factor for both chronic kidney disease and cardiovascular and cerebrovascular disorders. Blood pressure in the systemic arteries, which is consistently elevated, is its defining feature (93). Since atherosclerosis and hypertension are bidirectionally associated, inflammation is thought to be a trigger for hypertension, although the pathophysiology of hypertension varies by population or age (94). Patients with high blood pressure had higher serum IL-1β levels, according to earlier observations (95). These findings prompted research into the possible involvement of the NLRP3 inflammasome in hypertension from various causes.

To alleviate the symptoms of high blood pressure in preeclampsia patients, the first study to examine the relationship between NLRP3 and hypertension was conducted (96). Research has shown that sterile inflammation is involved in pathogenesis, but it is unclear whether the NLRP3 inflammasome is involved in pregnant NLRP3-/- and ASC-/-mice (97). In the NLRP3-/- mice, hypertension was avoided, but in the ASC-/-mice, there was no discernible reduction in blood pressure. These findings suggest that NLRP3 stimulates the progression of hypertension through a mechanism related to the inflammasome. Furthermore, NLRP3-/- mice presented decreased IL-6 but not IL-1β levels, indicating that NLRP3 plays a significant role in a wide range of inflammatory responses. Anti-NLRP3 therapy also benefits from reducing autoinflammation in the pathophysiology of hypertension (97).

Excessive exposure to salt has traditionally been regarded as a risk factor for hypertension. Although the precise process underlying salt-sensitive hypertension is still debated, oxidative stress and renal inflammation are two important potential causes of high blood pressure (98). As an inflammatory pathway closely linked to oxidative stress, NLRP3 and its associated upstream and downstream molecules play crucial roles in the progression of salt-sensitive hypertension. The paraventricular nucleus serves as a central hub for the endocrine system, regulating the dynamic equilibrium of the cardiovascular system. Suppressing NF-κB significantly reduces the production of proinflammatory cytokines and oxidative stress in the paraventricular nucleus, thereby mitigating salt-sensitive hypertension in a rat model (11). By activating the renin-angiotensin system and generating ROS in the paraventricular nucleus, direct inhibition of IL-1β, the primary proinflammatory cytokine in the NLRP3 inflammasome pathway, in addition to blocking NF-κB, also reduces hypertension (99).

In addition to causing cardiovascular disorders, hypertension is a major contributor to a number of cardiac injuries. The renin-angiotensin system is crucial for the common complication of hypertension, known as cardiac remodeling (100). Angiotensin II, often referred to as Ang II, is a constricting polypeptide that triggers stimulation of the renin-angiotensin system. A previous investigation revealed that injecting Ang II promoted the triggering of the NLRP3 inflammasome and the emission of inflammatory mediators. These cytokines contribute to the development of the inflammatory response implicated in myocardial fibrosis (101). EMD638683, a selective inhibitor that inhibits the activation of the NLRP3 inflammasome, was used to reduce cardiac fibrosis in a model of hypertension induced by Ang II in mice. However, hypertension did not appear to be impacted by the experiment. Additionally, hypertension can cause vascular remodeling and vascular endothelial cell impairment, which can worsen the progression of hypertension and create a vicious cycle of pathological blood pressure elevation. Vascular smooth muscle cell proliferation was alleviated by blocking NLRP3 with sinapine thiocyanate, whereas endothelial dysfunction was alleviated by blocking the upstream receptor TLR4 (102, 103). NLRP3 and its inflammasome work together to play significant roles in the onset and progression of hypertension. Treatment for hypertension appears to target NLRP3 in a novel way. However, clinical applications necessitate the development of a therapeutic protocol.

### Abdominal aortic aneurysm

Abdominal aortic aneurysm (AAA) is a serious vascular condition characterized by progressive pathological dilatation of the abdominal aortic wall. Although it usually has no symptoms, if it ruptures, it can be fatal. Research on pathophysiology and histopathology revealed that inflammatory cell infiltration, vascular smooth muscle cell (VSMC) loss, and aortic structural protein failure are always linked to the development of AAA (104). These findings suggest that the process of AAA involves the inflammatory response (105). It has been demonstrated that pyroptosis is crucial for vascular endothelium and VSMC damage. The NLRP3 inflammasome is a notable factor in the progression of AAA since it is the upstream trigger of pyroptosis.

Numerous studies have revealed the intricate mechanism involved at the molecular level. Wu and colleagues reported that when palmitic acid causes stress in human thoracic aortic SMCs, the NLRP3–caspase-1 inflammasome breaks down contractile fibers. Subsequent immunoprecipitation tests revealed that caspase-1 split the heavy chain in a straightforward manner after binding to the tropomyosin and myosin heavy chains in VSMCs and recombined with caspase-1. As a result, the ability of VSMCs to contract in a collagen matrix decreases. However, following the application of a caspase-1 blocking agent or siRNA-mediated knockdown of the NLRP3–caspase-1 inflammasome cascade, contractile function improved to a certain extent (106). These findings suggest that the NLRP3 inflammasome plays a mediating role in VSMC degradation of contractile proteins.

A study revealed that human VSMCs from AAA patients presented inflammatory characteristics and that their levels of NLRP3 and IL-1β mRNA and protein expression were greater than those in the control group (107). These findings confirmed that VSMC dysfunction and pyroptosis are caused by NLRP3-mediated inflammation, which in turn aids in the development of AAA.

The involvement of the inflammasome in AAA pathogenesis has been thoroughly demonstrated in mouse experiments. Additionally, reports have shown that IL-1 signaling plays a key role in AAA in a Kawasaki disease mouse model. Model mice in the control group experienced severe AAA. When exposed to the same stimuli, IL-1α-/-, IL-1β-/-, Casp1-/-, and Nlrp3-/- mice were effectively shielded from dilatation of the abdominal aorta. Neutralizing antibody injection produced similar protective outcomes to those seen in mice lacking certain genes (108). In a separate study, a reporter used MCC950 as a targeted NLRP3 inhibitor and investigated how wild-type mice provided with a diet rich in fat and cholesterol differed in the formation of AAAs. Eighty-seven percent of the mice experienced aortic dilatation following the intervention, whereas 98 percent of the mice in the control group experienced aortic dilatation. An apoptosis-associated molecule, matrix metallopeptidase 9 is triggered by the NLRP3 inflammasome. The findings demonstrated that mice treated with MCC950 had significantly lower levels of activated MMP-9, caspase-1, and IL-1β (109).

### Myocardial infarction

Myocardial infarction (MI), commonly referred to as a “heart attack,” represents a secondary symptom of insufficient blood flow to the myocardium that can cause hemodynamic decline, heart dysfunction, and even unexpected death (110). Myocardial ischemia symptoms are best treated with reperfusion therapy because of the limited capacity of the myocardium for regeneration. However, reperfusion is generally associated with high levels of inflammation, which leads to ischemia/reperfusion (I/R) injury and further deterioration of heart tissue. A major factor in the size of the infarction during the I/R process is the NLRP3 inflammasome.

The NLRP3 inflammasome may participate in I/R injury, as evidenced by the high expression level of ASC and the infiltration of inflammatory cells found in cardiac tissues from MI patients (26).

A previous study revealed that the NLRP3 inflammasome is involved in myocardial I/R damage. To create an I/R model, they transplanted bone marrow into wild-type mice to produce mice deficient in ASCs (ASC-/-mice). Compared with control mice, ASC-/-mice presented reduced ventricular remodeling and smaller infarcted areas, in accordance with previous results (26). Sandagers and associates reached a similar conclusion. Following MI surgery, mice presented increased mRNA expression levels of NLRP3, IL-1β, and IL-18 (111). Myocardial fibroblasts in the ex vivo Langendorff model presented an increase in the NLRP3 inflammasome, and mice lacking NLRP3 presented smaller infarction areas. Nonetheless, the fact that ASC-/- mice in the same study did not exhibit the anticipated result is still debatable. The possibility that ASCs also contribute to the formation of multiple other protective inflammasomes is a plausible explanation. Additionally, these findings demonstrated that the effects of NLRP3 are independent of inflammasomes (111). According to an *in vitro* study, PAMPs such as LPS, cellular ROS production, and potassium outflow can cause cardiac fibroblasts (rather than cardiomyocytes) to initiate the inflammatory response. Framework-related elements of the NLRP3 inflammasome were also found in cardiomyocytes from MI mice in a different experiment (112). However, they both went on to demonstrate how common NLRP3 is in inflammatory and cardiovascular cells.

In a nonreperfused AMI mouse model, NLRP3 inhibition did not reduce infarct size. Nevertheless, inhibition of the NLRP3 inflammasome in nonreperfused AMI prevents adverse remodeling, independent of infarct size (113). Inflammasome formation occurs in cardiomyocytes and fibroblasts for several days after AMI (112). IL-1β production is increased during AMI and modulates cardiac function and remodeling. IL-1 blockade with the recombinant human IL-1 receptor antagonist anakinra reduces cardiomyocyte death, preserves cardiac function and restores β-adrenergic receptor responsiveness in models of ischemic cardiomyopathy (114, 115).

### Dilated cardiomyopathy

According to epidemiological studies, dilated cardiomyopathy (DCM) is the primary cause of heart failure (HF) globally (116). DCM is defined by left ventricular enlargement and reduced contractility in the absence of abnormal loading conditions (117). Myocarditis, exposure to alcohol, medications, toxic substances, and metabolic and endocrine disorders are among the many causes of DCM and are classified into acquired and genetic types. NLRP3 is an essential component of cardiomyocyte pyroptosis, which is typically the primary cause.

The important role of caspase-1-dependent cardiomyocyte pyroptosis mediated by the NLRP3 inflammasome in DCM is supported by one experiment conducted in the heart (118). TUNEL, α-actinin, and active caspase-1 were triple-immunostained in nine human heart tissue samples. Compared with apoptotic cell death, pyroptotic cell death is more common in DCM patients. Compared with those in healthy controls, the plasma levels of IL-1β and IL-18, as well as the expression or phosphorylation of NF-κB were notably elevated in DCM patients. These findings indicate that the NLRP3 inflammasome has a substantial role in the progression of DCM (118).

NLRP3 not only triggers the inflammatory response but also promotes the development of fibrosis in DCM. In one study, the use of siRNA to knock down NLRP3 restored collagen I and III expression in the cardiac tissues of DCM model rats (119). Other studies have shown that NLRP3 may stimulate fibrosis and profibrosis via a number of pathways, including fibroblast cAMP signaling and the Smad (R-Smad) pathway (120). NLRP3 inhibition, either pharmacologicly or genetically, improves myocardial fibrosis (119). Fibrosis impairs the capacity of cardiomyocytes to contract, resulting in dysfunctional diastolic and systolic heart contractions.

### Diabetic cardiomyopathy

CVDs increase the mortality rate of people with diabetes mellitus (DM) (121); different CVDs are responsible for approximately half of the deaths of DM patients. The symptoms of diabetic cardiomyopathy (DCM) include interstitial fibrosis and diastolic and systolic dysfunction, along with an increase in left ventricular mass (122). Pyroptosis plays a crucial role in the development of DCM (119), and as previously mentioned (123), the NLRP3 inflammasome is crucial to this process. Hyperglycemia represents a potent stimulis for the NLRP3 inflammasome (124). In particular, in several cell types, high glucose levels induce ROS production and subsequent activation of nuclear factor kappa-light-chain enhancer of activated B cells (NF-κB) and TXNIP, thereby acting as priming and triggering signals to the inflammasome (125, 126). Although hyperglycemia is one of the factors that initiates the triggering of the NLRP3 inflammasome (127), the exact relationship between hyperglycemia and the inflammatory reaction has not been fully investigated.

Type 1 diabetes (T1D) and type 2 diabetes (T2D) are the two subtypes of DM. While T2D can also cause hyperlipidemia, both T1D and T2D cause hyperglycemia. Notable indicators of NLRP3 inflammation in DCM include lipotoxicity and glucotoxicity, which involve both the priming and activation phases. High blood glucose and lipid levels have been shown in several studies to increase ROS overexpression (128), which in turn activates the NF-κB signaling pathway and stimulates the transcription of NLRP3, pro-IL-1β, and pro-IL-18 (129). The second signal in NLRP3 activation is thioredoxin interacting/inhibiting protein (TXNIP), which attaches directly to NLRP3 to alter its oligomerization (130). Hyperglycemia-induced ROS production may increase TXNIP expression, allowing its activation via a different pathway. Hyperglycemia and hyperlipidemia, including fatty acids, facilitate this process via bypass pathways in addition to the traditional pathway of inflammation. This exacerbates mitochondrial oxidative stress and other proinflammatory cytokines, which consequently triggers the development of the NLRP3 inflammasome. The activation of the NLRP3 inflammasome is facilitated by cytosolic Ca2+ along with the traditional ROS-mediated activation pathway (131). Sarcoplasmic/endoplasmic reticulum calcium ATPase 2 (SERCA2a) is a crucial enzymatic protein that keeps the endoplasmic reticulum and cytoplasm accessible. Its malfunction causes a disorder of Ca2+ transport, which in turn triggers NLRP3 activation and pyroptosis. In T1D rats, SERCA2a levels decrease in tandem with a decrease in sarcoplasmic reticulum function (132). Similarly, data from T2D mice revealed a decrease in SERCA2a expression. However, in contraction experiments, increased SERCA2a expression in DM cardiomyocytes significantly improved contractile function.

### Heart failure

Heart failure (HF) is the final stage of nearly all severe cardiovascular diseases and is caused by a variety of structural or functional abnormalities of the heart (133). It is widely acknowledged that cardiac remodeling, in which inflammatory mediators have a substantial impact, is the underlying mechanism of heart failure (134). Myocardial damage caused by pyroptosis and cardiac fibroblast activation are two physiological processes that have been identified as the primary steps in the remodeling process.

Previous studies have thoroughly examined traditional activators such as ROS and oxLDL-C. Another potential mechanism of apoptosis induced by inflammatory cytokines involves the discovery of novel physiological functions of NO. Vascular smooth muscles or other pathways are relaxed by low amounts of NO produced by constitutive NO synthase, which protects cardiomyocytes (135). However, excessively high NO levels produced by enzymes such as inducible nitric oxide synthase (iNOS) lead to cardiac injury and facilitate leukocyte-endothelial interactions (136). The overexpression of iNOS is promoted by IL-18, whereas IL-1β is a potent stimulator of iNOS (137, 138). The cleavage of caspase-1 on the NLRP3 inflammasome is necessary for the maturation of both proteins (139, 140). In addition to the excessive production of NO and the ensuing apoptosis and reconfiguration, an elevated iNOS level also causes the generation of tiny, uncharged NO molecules. NO- molecules have functions similar to those of reactive nitrogen species (ROS) and can change into RNS (140).

In addition to NO-induced death facilitated by the IL-1 family, other inflammatory cytokines also contribute to the development of heart failure. One important cytokine that promotes inflammation and causes cell hypertrophy and apoptosis is tumor necrosis factor α (TNF-α) (141, 142). In a healthy heart, TNF-α is not present. By reducing intracellular Ca2+ release, TNF-α, which is present in cardiomyocytes in HF animal models, reduces cardiac contractility (143). One byproduct of NLRP3 activation is IL-18, which aids in the synthesis of TNF-α. Conversely, TNF-α can stimulate the NF-κB pathway, which in turn promotes the transcription of NLRP3 and IL-1. This interaction exacerbates inflammatory damage to myocardial tissue.

The primary mechanism involved in ventricle remodeling is fibrosis. In two-thirds of heart tissue, fibroblasts are essential for the development of ventricle fibrosis (26). Since the NLRP3 inflammasome is recognized as being significantly triggered by the generation of ROS and the outflow of K+ from cardiac fibroblasts, the absence of oxygen encourages these processes (144). Following hypoxia, the NLRP3 inflammasome is the first DAMP sensor. Following myocardial ischemia damage, fibroblasts cause and amplify inflammatory impairment by activating the NLRP3 inflammasome. Hypoxia causes cardiac fibroblasts to exhibit a fibrogenic phenotype in addition to an inflammatory response, which causes myofibroblasts to transdifferentiate and produce more collagen (144). Myocardial fibrosis, inocyte proliferation, and ventricle remodeling are the ultimate results of these processes. Through its structure, NLRP3 has also been found in recent studies to promote cardiac fibroblast differentiation, which is independent of inflammasomes (145). Profibrotic gene expression is ultimately caused by the regulation of mtROS expression levels by NLRP3 and the enhancement of the R-Smad signaling pathway. This finding offers a new way for myocardial fibrosis to occur.

### Calcific aortic valve disease

Calcific aortic valve disease (CAVD) causes aortic stenosis (AS) and the adaptive reactions that follow, such as left ventricular hypertrophy and heart failure (146). The only effective treatment for CAVD is aortic valve replacement surgery or intervention (147). However, these costly interventions are often delayed until the late stages of the disease. Given the current landscape, next-generation therapies for CAVD are needed to improve patient outcome and quality of life.

Aortic valve calcification was first attributed to degenerative osteogenic processes and passive substance deposition, similar to the pathological mechanism of atherogenesis. Recent research, however, has shown that the precise underlying cause of CAVD may involve positive processes such as osteoblastic differentiation, lipoprotein deposition, and chronic inflammation (148, 149). However, a comprehensive and detailed explanation of the underlying development of the inflammatory response in CAVD is still lacking. Heart muscle cells, smooth muscle cells, valve endothelial cells (VECs), and valve interstitial cells (VICs) make up the human aortic valve. Compared with other mesenchymal cell types in other organs, VICs are the most prevalent type of cell (150). Quiescent VICs (qVICs), activated VICs (aVICs), progenitor VICs (pVICs), osteoblastic VICs (obVICs), and embryonic progenitor endothelial/mesenchymal cells are the five subtypes into which they are typically separated according to their distinct functions and levels of gene expression (151). They can change from one form to another and are not fixed. Under normal circumstances, VICs typically remain dormant to preserve normal valve function. The inflammatory response and cytokines can convert qVICs into aVICs when valve damage occurs (149). aVICs function similarly to the mesenchymal stem cells involved in tissue repair and regeneration, differentiate into cells of the myofibroblast type, and, upon activation, express the marker alpha-smooth muscle actin (αSMA) (152). The activation of aVICs is followed by cell growth and extracellular matrix reorganization, which disrupts the architecture of the valve and causes cusp thickening or fibrosis. This leads to a vicious cycle whereby damage to qVICs and VECs becomes worse, making it more difficult to return to physiological conditions. Following the accumulation of impairments, the aortic valve calcifies, activates obVICs, and undergoes osteogenic differentiation.

Inflammation did not receive much attention at first. However, an aberrant phenomenon has given rise to a new mechanism of CAVD: calcific valves contain inflammatory cells that are not present in normal valves, including mast cells, T lymphocytes, and macrophages (153, 154). These findings suggest that one of the main causes of CAVD may be inflammation. A recent study suggested that NLRP3 may be involved (155). Inhibition of the NLRP3 inflammasome by Th1 cells protects against VIC calcification as a negative feedback mechanism of adaptive immunity toward innate immunity (156). Blocking the NLRP3 inflammasome also reduces osteogenic calcification and macrophage polarization in a mouse model of calcified aortic valve stenosis (157). The detailed process by which the NLRP3 inflammasome facilitates the progression of CAVD has not yet been fully investigated. The bark of coniferous trees and honeybees naturally contains a polyphenolic compound called caffeic acid phenethyl ester (CAPE) (158), which inhibits the development of calcified nodules. Additionally, the addition of CAPE prevents NF-κB from being phosphorylated (155). Human aortic valvular interstitial cells were cultivated to assess how CAPE affects osteogenesis and NF-κB pathway activation. CAPE prevents NF-κB signaling pathway activation, inhibits the transcription of NLRP3, and strongly suppresses the ensuing inflammation (159).

For CAVD, there are currently no available medication treatment options. Nonetheless, investigations into the fundamental pathophysiology of CAVD have provided fresh insights into potential treatments. CY-09 exerted a protective effect on calcified aortic valve stenosis in addition to blocking NLRP3 with CAPE (157). Furthermore, the results from 2518 patients who underwent TAVR revealed that statin treatment resulted in markedly decreased BHV calcification in studies on bioprosthetic heart valves (160). Because statins reduce inflammation, immunosuppressive treatments that target the NLRP3 inflammasome or its byproducts are potential treatment options for valve calcification. Early anti-inflammatory therapy may prevent the progression of CAVD. The exact relationship between CAVD and the inflammatory reaction has not been fully investigated.

## NLRP3 inflammasome Inhibitors

**Figure 2:**
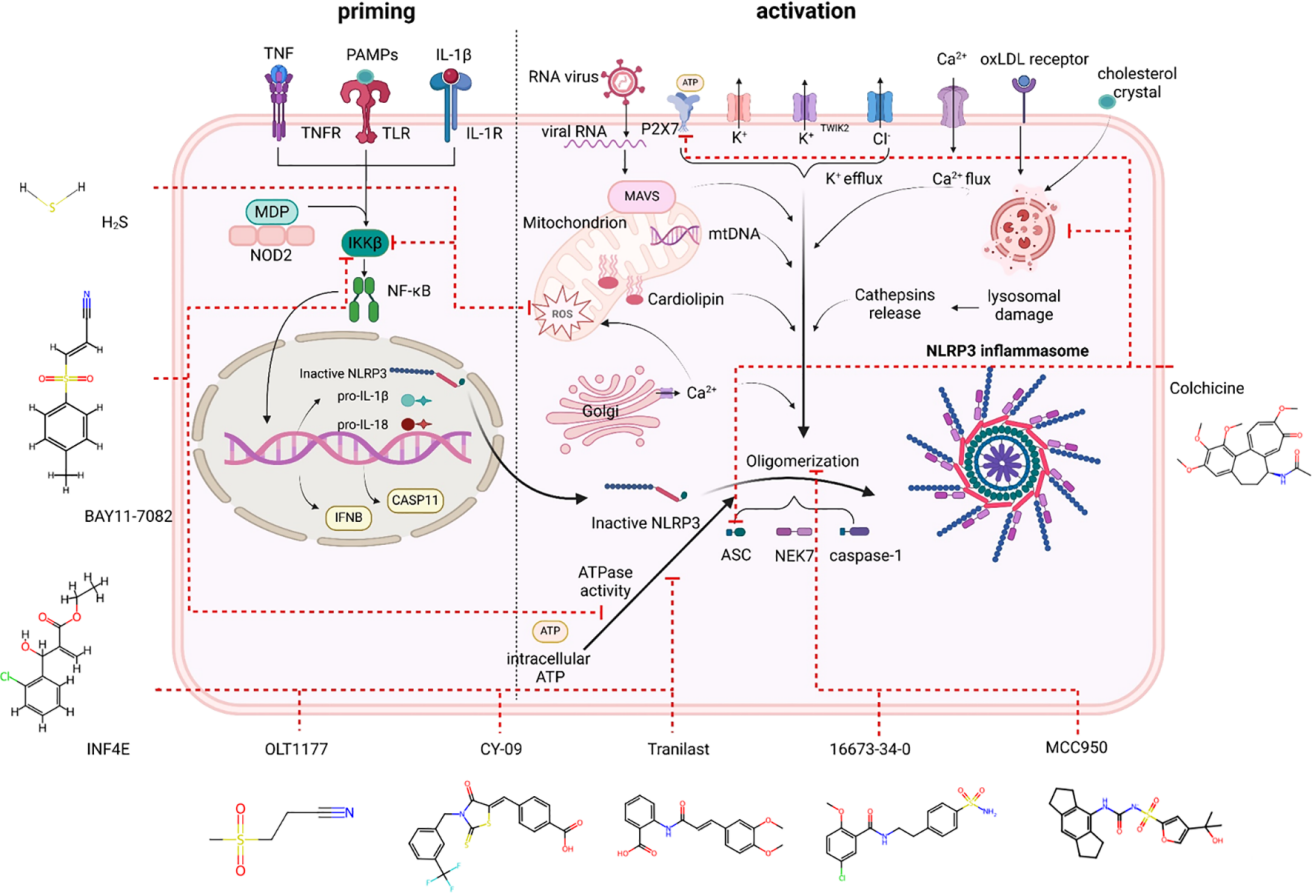
NLRP3 inhibitors under clinical development in cardiovascular diseases: chemical structure, molecular target and mechanisms of action.

Targeting the core components of the NLRP3 inflammasome can prevent pyroptosis, which is unaffected by the inhibition of IL-β or IL-18. Cellular assays often involve costimulating cells with lipopolysaccharide (LPS) and ATP, or alternative stimuli such as nigericin, cholesterol crystals, and monosodium urate (MSU) crystals, as well as *in vivo*, and the effectiveness of NLRP3 inflammasome inhibitors has been investigated. Animal models of cardiovascular disease have been used to test the majority of the compounds reviewed here. **Figure 2** shows several NLRP3 inflammasome inhibitors that have been investigated in the cardiovascular system.

### Glyburide, 16673-34-0, and JC-124

Glyburide, also known as glibencamide, is a sulfonylurea that has been authorized for the management of type II diabetes mellitus (161). Inhibiting the ATP-sensitive potassium channel (KATP) in pancreatic beta cells stimulates the release of insulin (162). Glyburide was the first chemical compound identified as an inhibitor of the NLRP3 inflammasome in bone marrow-derived macrophages stimulated with LPS and ATP (163). Nevertheless, the high dosages required for the anti-inflammasome effects of glyburide cause severe hypoglycemia, which may restrict its use as an anti-inflammatory medication *in vivo* (28). The inhibitory activity of the NLRP3 inflammasome does not depend on the cyclohexylurea moiety, which is associated with insulin secretion (163). This crucial piece of knowledge resulted in the creation of 16673-34-0, an orally active substance that can particularly suppress the NLRP3 inflammasome without influencing glucose catabolism despite lacking the cyclohexylurea moiety (163). Several cardiac injury models have been used to test the effects of 16673-34-0 (28, 163). Without influencing glucose levels, 16673-34-0 can decrease cardiac injury, as indicated by infarct size and cardiac troponin I levels in mice (28). On the basis of 16673-34-0, new compounds were created, such as JC-124 (an N-Me sulfonamide analog of 16673-34-0). When JC-124 was administered intraperitoneally to mice subjected to ischemia and reperfusion, it was able to decrease the size of the infarct and the levels of plasma troponin I (164).

16673-34-0 enhanced cardiac function in a nonreperfused model of ischemia without decreasing the size of the infarct (28). A previous study reported that administering 16673-34-0 to mice that experienced ischemia decreased ischemic heart damage and enhanced postreanimation cardiac function *in vivo* (165). 16673-34-0 demonstrated the same effectiveness in models of nonischemic cardiac injury (28). Indeed, 16673-34-0 administered intraperitoneally enhanced cardiac function and decreased interstitial fibrosis in mice administered doxorubicin (28). Systolic and diastolic dysfunction were prevented in a murine model of cardiomyopathy induced by a Western diet when 16673-34-0 was administered in the feed at a concentration of 100 mg/kg (166). 16673-34-0 decreased pericardial thickening and pericardial effusion in mice with experimental pericarditis (91).

### MCC950

In 2001, MCC950, also referred to as CP-456,773 or CRID3, was first reported to inhibit IL-1β processing along with other compounds containing diarylsulfonylurea (167). Coll et al. provided further characterization demonstrating strong *in vitro* and *in vivo* inhibition of NLRP3 (168). This small molecule effectively attaches to NLRP3, inhibiting its ATPase activity and thereby hindering ASC oligomerization and the release of IL-1β (168, 169). MCC950 cannot inhibit AIM2, NLRP1, or NLRC4 (168–170). MCC950 is unable to effectively restrain the cryopyrin-associated periodic syndrome forms of NLRP3 because, according to a recent study, the NACHT domain of NLRP3 serves as the molecular target of diarylsulfonylurea suppressors (109).

MCC950 has been investigated in a mouse model of atherosclerosis. The development of atherosclerosis is strongly decreased by MCC950 at a daily dosage of 10 mg/kg (171). Acute myocardial infarction models in both small and large animals have demonstrated the positive effects of MCC950. Pigs that received a 7-day treatment presented improvements in myocardial IL-1β levels, infarct size, reduction in neutrophil infiltration, and preservation of arterial function (172). In mice subjected to permanent coronary artery occlusion, MCC950 improved cardiac remodeling by lowering myocardial fibrosis, caspase-1 activation, inflammatory cell infiltration, and IL-18 and IL-1β expression levels (173). MCC950 can also effectively reduce inflammation when it is administered via hydrolytic microspheres to the ischemic area. After receiving MCC950, mice that underwent potassium-induced murine cardiac arrest and subsequent cardiopulmonary resuscitation also presented improved neurologic function and survival (109).

A previous study demonstrated that MCC950 (10 mg/kg) can successfully reduce myocardial fibrosis and IL-1β levels when given three times to patients with hypertension caused by angiotensin II infusion (174). The blood pressure of mice with established hypertension decreased, and renal inflammation was reduced after 25 days of MCC950 (10 mg/kg) treatment. Aortic dilation, dissection, and rupture in the thoracic and abdominal aortic segments of mice treated with a high-fat, high-cholesterol diet and Ang II were markedly inhibited by MCC950 (175).

When MCC950 (10 mg/kg) was given to mice three times weekly for eight weeks in a mouse model of postmenopausal cardiovascular disease, it increased systolic and diastolic function, decreased atrial natriuretic peptide and BNP mRNA expression levels, and limited hypertrophic remodeling (176). The protective effects of MCC950 on cardiac tissues in NLRP3−/− mice were replicated by prolonged administration of MCC950 in obesogenic mice. After 15 weeks, MCC950 decreased heart apoptosis and increased autophagy flux in these mice (177). When an excessive amount of AngII is infused into cardiac tissue, MCC950 decreases myocardial remodeling (174).

### Bay 11-7082

Bay 11-7082, a phenyl vinyl sulfone that blocks the inhibitor of kappa B kinase (IKK) β, was first discovered to be an inhibitor of the NF-κB signaling pathway (178). The NLRP3 inflammasome can be inhibited by Bay 11-7082, whereas the other inflammasomes (NLRP1 and NLRC4) are unaffected. Some NLRP3 inhibition caused by Bay 11-7082 does not depend on NF-κB-mediated priming inhibition. The cysteine residues of the NLRP3 ATPase region are alkylated by Bay 11-7082 (178). The intraperitoneal administration of Bay 11-7082 10 min prior to reperfusion effectively reduced infarct size, cardiomyocyte apoptosis, and the inflammatory response in a murine model of ischemia/reperfusion (179). Pretreatment with Bay 11-7082 maintained cardiac function and reduced infarct size, cardiac fibrosis, and apoptosis in a rat model of myocardial ischemia-reperfusion (180). Comparable outcomes were noted in diabetic rats, where Bay 11-7082 decreased pyroptosis, NLRP3 inflammasome stimulation, caspase-1 activity, and IL-1β production to mitigate myocardial damage after ischemia-reperfusion (181). However, the effects of Bay 11-7082, which rely on NF-κB pathway inhibition, are challenging to distinguish *in vivo*.

### OLT1177

The NLRP3 inflammasome is specifically inhibited by the oral beta-sulfonyl nitrile molecule OLT1177 (182). It has no effect on AIM2 or NLRC4, but it decreases the release of IL-18 and IL-1β. By blocking NLRP3 oligomerization, OLT1177 stops the downstream cascade from being activated, and the NLRP3–ASC interaction does not occur. Through direct interaction, OLT1177 inhibits the ATPase activity of NLRP3 (182). OLT1177 consistently inhibits NLRP3 activation in several preclinical models of inflammatory disease (182–184). Furthermore, OLT1177 decreased the release of cytokines in mononuclear cells taken from CAPS patients, where constitutively active mutants of NLRP3 spontaneously secreted IL-1β and IL-18 without infection or tissue damage (182). OLT1177 maintained cardiac function 24 hours and 7 days postreperfusion and decreased the infarct size in a dose-dependent manner in an animal model of myocardial ischemia-reperfusion (185). In a model involving permanent occlusion of the coronary arteries, OLT1177 also enhanced ventricular function. Since OLT1177 has demonstrated efficacy when administered 60 minutes after rep, the same study demonstrated its applicability in a clinically relevant scenario.

OLT1177 is undergoing clinical trials. OLT1177 proved to be reliable and potent for alleviating target joint pain in individuals with gout, a condition that depends on NLRP3 inflammasome activation, in an open-label phase 2A study (186). OLT1177 was deemed safe, and at the highest dose administered, it was associated with an increase in the left ventricular ejection fraction and treadmill exercise duration after 14 days in a pilot phase-1B, double-blind trial involving patients with heart failure and a reduced ejection fraction (187).

### INF4E

The antipyroptotic properties of a collection of α,β-unsaturated carbonyl or cyano compound derivatives were screened and synthesized (29). Because of their reactive Michael acceptor fragment, these substances inhibit NLRP3. Because of its capacity to suppress NLRP3 ATPase activity and caspase-1 activation, INF4E was selected among these substances (29). In ex vivo studies, rat hearts perfused with a Langendorff apparatus and subjected to 30 minutes of ischemia followed by 20 or 60 minutes of reperfusion presented improved left ventricular pressure, decreased lactate dehydrogenase, and smaller infarcts after pretreatment with INF4E. In addition, INF4E treatment stimulated the protective reperfusion injury salvage kinase (RISK) signaling pathway, enhanced mitochondrial function, and decreased the expression level of the NLRP3 inflammasome in these hearts (188). Owing to the possible cytotoxicity of this compound, the same group created additional compounds in a more recent study that share a sulfonamide or sulfonylurea portion and the Michael acceptor moiety of INF4E (189). INF58 is the most promising of these substances, but its cardioprotective properties have proven to be effective.

### Tranilast

Trinilast (N-[30-40-dimethoxycinnamonyl]-anthranilic acid, TR), an analog of a tryptophan metabolite, has received clinical approval for treating several allergic conditions (190). Although it has been demonstrated to decrease collagen synthesis, the exact mechanical process underlying this effect is unclear. Tranilast was recently discovered to be an inhibitor of the NLRP3 inflammasome with no discernible effects on AIM2 or NLRC4 (191). It has been demonstrated to bind directly to the NACHT domain of NLRP3, blocking it from oligomerizing without assisting in NLRP3 ATPase activity. Additionally, its suppressive impact is unaffected by upstream signal transduction, including the generation of ROS, ion efflux, and mitochondrial dysfunction (191). Tranilast has beneficial effects on diseases linked to the NLRP3 inflammasome in animal models of gout, type 2 diabetes, and CAPS. More recently, tranilast increased NLRP3 ubiquitination in two murine models of atherosclerosis, which inhibited the assembly of the NLRP3 inflammasome and, as a result, slowed the development and spread of atherosclerotic plaques (192). Tranilast has been shown to have positive effects on cardiac fibrosis and remodeling in multiple animals since its discovery as an NLRP3 inhibitor (187).

### CY-09

CY-09, a newly created NLRP3 inhibitor, blocks NLRP3 assembly and ATPase activity by directly attaching to the ATP-binding motif of the NACHT domain. Animal models of type 2 diabetes mellitus and CAPS have been used to test its therapeutic efficacy (193). In a diabetic stroke mouse model, CY-09 was effective in preventing the cardiac dysfunction linked to diabetic ischemic stroke (194).

Clinical experience with targeted NLRP3 inflammasome inhibitors is limited. Indirect evidence of the central role of the NLRP3 inflammasome is derived from nonselective NLRP3 inhibitors, such as colchicine, or blockers of IL-1 and IL-18.

## Potential therapies associated with the NLRP3 inflammasome

Numerous studies have been conducted to investigate potential treatment approaches that target the inflammatory response since the function of the NLRP3 inflammasome in cardiovascular diseases has been increasingly validated (195). Suppressing the NLRP3 inflammasome or its upstream modulators and downstream mediators has garnered much attention because NLRP3 is a novel mediator of the inflammatory response. In recent years, a number of inhibitors that target the NLRP3 inflammasome and IL-1β have shown favorable results in clinical trials (**Table 1**).

**Table 1:** Overview of completed clinical trials targeting the NLRP3 inflammasome in cardiovascular diseases.

Clinical trials	Intervention	Targeted diseases	Number of patients	Major outcomes	Major serious adverse outcomes	Sponsor	Results first posted year	PMID or NCT number
Interleukin-1 (IL-1) blockade in acute myocardial infarction (VCU-ART3)	Anakinra	Acute myocardial infarction	99	Acute phase response (CRP Levels)	Infection	Virginia Commonwealth University	2019	NCT01950299
Anakinra to Prevent Adverse post-infarction Remodeling (2)	Anakinra	Acute myocardial infarction, heart failure	30	Change in left ventricular end-systolic volume indices	Serious infection	Virginia Commonwealth University	2016	NCT01175018
Treatment of Acute Pericarditis With Anakinra	Anakinra	Acute Pericarditis	5	Acute Pericarditis	Thrombophlebitis	Virginia Commonwealth University	2021	NCT03224585
Interleukin-1 blockade in recently decompensated heart failure	Anakinra	Heart Failure	60	Interval changes in peak oxygen consumption (vo2)	Serious infection, acute kidney injury	Virginia Commonwealth University	2017	NCT01936909
Interleukin-1 blockade in HF with preserved EF	Anakinra	Heart failure	31	Heart failure	Acute decompensated heart failure	Virginia Commonwealth University	2018	NCT02173548
Interleukin (IL)-1 blockade in acute heart failure (Anakinra ADHF)	Anakinra	Heart failure	30	Plasma C reactive protein levels	Serious infection/sepsis	Virginia Commonwealth University	2016	NCT01936844
Anakinra to prevent post-infarction remodeling (VCU-ART)	Anakinra	ST segment elevation acute myocardial infarction	10	Change in end-systolic volume indices	–	Virginia Commonwealth University	2010	NCT00789724
Canakinumab anti-inflflammatory thrombosis outcome study	Canakinumab	Cardiovascular events	10,061	non-fatal MI, non-fatal stroke, cardiac death	infection/sepsis	Novartis Pharmaceuticals	2,017	28845751
Safety and effectiveness on vascular structure and function of ACZ885 in atherosclerosis and either T2DM or IGT patients	Canakinumab	Atherosclerosis	189	Change in aortic distensibility and plaque burden	Infections, nervous system disorders	Novartis Pharmaceuticals	2015	NCT00995930
ACZ885 for the treatment of abdominal aortic aneurysm	Canakinumab	Abdominal aortic aneurysm	65	Change from baseline in abdominal aortic aneurysm (AAA) size per year	Hip fracture	Novartis Pharmaceuticals	2016	NCT02007252
Low-dose colchicine 2	Colchicine	Chronic, stable coronary artery disease	5,522	Cardiovascular events	non–CVDrelated deaths	Heart Research Institute of Western Australia	2020	32865380
Colchicine cardiovascular outcomes trial (COLCOT)	Colchicine	Recent acute myocardial infarction	4,745	Ischemic cardiovascular events	Pneumonia	Montreal Heart Institute	2020	NCT02551094
Low-dose colchicine	Colchicine	Stable coronary artery disease	532	Cardiovascular events	–	Heart Research Institute of Western Australia	2013	23265346
Colchicine for left ventricular infarct size treatment in acute myocardial infarction	Colchicine	First stable ST segment elevation acute myocardial infarction	192	Infarct size, left ventricular remodeling, left ventricular end-diastolic volume	Left ventricular thrombus, gastrointestinal adverse events	French Ministry of Health	2021	34420373
Study to assess the efficacy and safety of rilonacept treatment in participants with recurrent pericarditis	Rilonacept	Recurrent pericarditis	86	Time to pericarditis recurrence	Cardiac flutter, squamous cell carcinoma	Kiniksa Pharmaceuticals (UK), Ltd.	2021	NCT03737110
Rilonacept to improve artery function in patients with atherosclerosis	Rilonacept	Atherosclerosis	10	C-reactive protein levels	Upper respiratory infection	National Heart, Lung, and Blood Institute (NHLBI)	2010	NCT00417417

### Anakinra

Anakinra is a gene recombination-prepared selective IL-1 receptor antagonist that inhibits the action of both IL-1α and IL-1β. Current clinical trials of anakinra in CVD patients are concerned primarily with myocardial infarction, heart failure, and pericarditis. A pilot study evaluating the immediate efficacy of anakinra in individuals with acute pericarditis was published in 2021 (196). In patients given 100 mg of anakinra, the results demonstrated a significant reduction in pain scores. The trial was stopped because of remarkable results in the first 24 hours, which showed that anakinra was a promising treatment for pericarditis.

Three studies on anakinra for heart failure treatment comprehensively evaluated changes in plasma C-reactive protein levels, ventilatory efficiency, and variations in peak oxygen consumption (VO2) over time (197, 198). Anakinra was found to have a positive effect on systemic inflammation, and NT-proBNP showed a positive trend following anakinra. The ejection fraction of anakinra-treated volunteers was maintained or increased, even though their VO2 did not appear to be impacted during the trial.

Furthermore, the potential of using anakinra to treat MI was also assessed. The safety and effectiveness of anakinra in treating acute myocardial infarction were thoroughly assessed in one group through three trial phases (VCU-ART) (198–200). A total of 139 patients participated in the trials, and the findings showed that anakinra markedly reduced the systemic inflammatory response after myocardial infarction. The treatment group presented a lower risk of heart failure following myocardial infarction, despite the apparent neutral effect of anakinra on improving the left ventricular ejection fraction and recurrent ischemic events.

Although anakinra has been approved for treating a number of immune-related conditions and has potential for use in anti-inflammatory therapy, none of its treatments have been approved for treating cardiovascular diseases such as pericarditis. Further comprehensive clinical trials are needed to confirm the safe application of anakinra in treating cardiovascular diseases.

### Colchicine

Colchicine is a common anti-inflammatory medication used to treat gout and pericarditis. It prevents ASC and NLRP3 from forming microtubes and from moving subcellularly, and it also prevents the inflammasome from assembling. Compared with other inflammasome-targeting agent blockers of IL-1 or IL-18, colchicine is a nonselective NLRP3 inhibitor. The potential of using colchicine to treat other CVDs is still being investigated, although it is acknowledged as the primary treatment for pericarditis. Many extensive clinical trials have been carried out in an effort to treat various CVDs because colchicine is a safe and affordable anti-inflammatory medication.

In the low-dose colchicine (LoDoCo) study, 532 patients with stable coronary artery disease were randomly assigned to either the colchicine or placebo group. This is the first major trial to focus on cardiovascular outcomes (201).The group treated with colchicine presented a significantly reduced risk of cardiovascular events during the median 3-year follow-up period for all volunteers. This conclusion was also supported by the LoDoCo2 trial, which randomized 5,522 volunteers and reported that colchicine treatment reduced the main composite endpoints by 31% (202).

In recent tests, the potential of colchicine for treating MI has attracted much attention. According to the 2020 Unblinded Colchicine Cardiovascular Outcomes Trial (COLCOT), the group receiving colchicine had a lower incidence of coronary and cerebral atherothrombotic events than did the placebo group (203). The first stable ST-segment elevation acute MI was the subject of a subsequent, comparatively small study. However, the trial’s findings revealed no change in the size of the infarct over the course of five days and three months following MI (204). This randomized pilot trial was limited by a small population size and was not powered to demonstrate differences in long-term hard clinical end points; therefore, these results should be generalized with caution. Colchicine may be a promising treatment for multiple CVDs, according to the evidence above; however, additional research is needed to confirm the treatment’s impact on a given CVD. The prevention of CVDs is another area of ongoing research. These findings may lead to a new understanding of the use of colchicine in CVD treatment.

### Canakinumab

The monoclonal antibody canakinumab can neutralize IL-1β directly. Canakinumab’s Canakinumab Anti-Inflammatory Thrombosis Outcomes Study (CANTOS) is the most discussed clinical trial (205). A total of 17,200 patients who experienced acute MI for more than 29 days prior to the study were randomly assigned to the trial, which started in late 2011. A total of 10,061 volunteers were ultimately enrolled for additional research. Even after receiving standard secondary prevention treatments consistently, the chosen volunteers’ hsCRP levels were higher than 2–0 mg/L. To determine the ideal dosage of canakinumab, the CANTOS trial established three subcutaneous injection doses: 50, 150, and 300 mg every three months. Nonfatal MI, nonfatal stroke, or cardiovascular death was designated the first endpoint.

According to data released in 2017, patients receiving 150 mg of canakinumab every three months experienced a 15% lower risk of recurrence than those receiving a placebo. This result is statistically notable, as canakinumab outperforms other secondary prevention therapies, offering a viable complement to standard statin treatment. Furthermore, the initial large-scale, double-blind, randomized controlled clinical trial that focused on a single inflammatory cytokine in the course of CVD was the CANTOS. The CANTOS results confirmed the potential to improve the outcome of patients post-MI by targeting the function or production of IL-1β, highlighting the crucial role of IL-1β in the progression of cardiovascular diseases.

In addition to the anticipated results in reducing cardiovascular diseases, the CANTOS trial delivered an unexpected finding: the overall mortality rate remained unchanged, despite the inhibition of IL-1β leading to a weakened innate immune system. This surprising outcome was attributed to a decrease in cancer-related deaths. In particular, deaths from lung cancer were significantly lower in the CANTOS population (179 participants). One logical explanation is that IL-1β contributes to the development of an inflammatory tumor microenvironment, promoting carcinogenesis in various types of cancer. The decrease in lung cancer mortality observed in the CANTOS trial suggests that IL-1β-directed therapies could benefit patients with specific types of cancer (206).

In addition to the CANTOS, 189 volunteers participated in a different trial that investigated the viability of blocking IL-1β in the management of atherosclerosis. Canakinumab successfully decreased the levels of inflammatory markers such as hsCRP and IL-6, but the trial did not reveal any statistically significant alterations in the mean carotid wall area or aortic distensibility. In addition, lipoprotein levels were lower than those in the placebo group. However, canakinumab increased triglyceride and total cholesterol levels. The use of canakinumab to treat abdominal aortic aneurysms has also been investigated, in addition to MI and atherosclerosis. The aortic diameter of volunteers receiving canakinumab treatment decreased nonsignificantly. However, this trial ended in the third interim analysis because it was ineffective and futile. There is ongoing doubt regarding the efficacy and validity of canakinumab treatment for abdominal aortic aneurysms.

As a promising IL-1 pathway inhibitor, canakinumab is used to treat cardiovascular diseases. However, to provide evidence in favor of the use of canakinumab in CVD, more extensive and thoroughly reviewed clinical trials are needed.

### Rilonacept

Rilonacept is a dimeric fusion protein that consists of the Fc portion of IgG1 and the extracellular portion of the IL-1 receptor. Unlike canakinumab, which can neutralize IL-1β directly, rilonacept stops IL-1β and IL-1α from interacting with receptors on the cell surface. Although research on the use of rilonacept to treat CVD has received early attention, clinical trial results are scarce. The effect of rilonacept on atherosclerosis was examined in a small-scale trial in 2010 that involved ten volunteers (207). Compared with that in the placebo group, the CRP level in the treatment group was lower. However, the difference was not statistically significant between the groups. In a 2019 trial on recurrent pericarditis, rilonacept was shown to be an effective treatment for pericarditis pain and inflammation (208). While there are no growing safety concerns, the dependence on corticosteroids has been effectively reduced or eliminated, and health-related quality of life has increased. The United States authorized the use of rilonacept in 2021. It is the first medica the role of immunology tion approved by the FDA to stop recurrent pericarditis in patients older than twelve years.

The FDA has not yet approved any specific NLRP3 inhibitor for use in CVD therapies, which is one of the main obstacles to the advancement of NLRP3 pathway-targeting treatment. Some drugs are not available for clinical use, and data on cardiovascular outcomes are lacking. Further large-scale studies are necessary to draw definitive clinical conclusions. However, several clinical trials, including those involving dapansutrile and tranilast, have been carried out to determine the effectiveness of NLRP3 inhibitors in CVD treatment. The results showed that treating CVDs with NLRP3 inhibitors was safe and well tolerated by volunteers, and the treatment showed promising efficacy (186). Individuals will gain if practical and targeted treatments are created and implemented.

## Conclusion

NLRP3, a key immune system-related PRR, is activated by a variety of PAMPs and DAMPs. IL-1β and IL-18 are produced, cleaved, and released when the NLRP3 inflammasome is formed, resulting in low-grade chronic inflammatory responses. Numerous CVDs are influenced by inflammation, according to recent research. In-depth research has been conducted on the mechanisms involved. These factors can be broadly summarized as follows: a variety of triggers initiate the transcription process through the NF-κB pathway or NLRP3 inflammasome assembly, which causes IL-1β to be produced, mature, and released; IL-1β then stimulates transcription through IL-1R. In related studies, NLRP3 silencing, NF-κB pathway blockade, and IL-1β binding all produced favorable outcomes for CVD prevention and control. Additionally, anti-inflammatory therapy targeting IL-1β shows promise for patients with specific cancers, particularly lung cancer. Direct inhibition of the NLRP3 inflammasome appears to have the fewest adverse effects among the various inhibition schemes, as anticipated.

## References

[1] Benjamin EJ Virani SS Callaway CW Chamberlain AM Chang AR Cheng S. Heart disease and stroke statistics-2018 update: A report from the american heart association. Circulation. (2018) 137:e67–e492. doi: 10.1161/CIR.0000000000000558 29386200

[2] Mayerl C Lukasser M Sedivy R Niederegger H Seiler R Wick G. Atherosclerosis research from past to present–on the track of two pathologists with opposing views, Carl von Rokitansky and Rudolf Virchow. Virchows Archiv: an Int J Pathol. (2006) 449:96–103. doi: 10.1007/s00428-006-0176-7 16612625

[3] Abbate A Toldo S Marchetti C Kron J Van Tassell BW Dinarello CA. Interleukin-1 and the inflammasome as therapeutic targets in cardiovascular disease. Circ Res. (2020) 126:1260–80. doi: 10.1161/CIRCRESAHA.120.315937 PMC8760628 32324502

[4] Mauro AG Bonaventura A Mezzaroma E Quader M Toldo S. NLRP3 inflammasome in acute myocardial infarction. J Cardiovasc Pharmacol. (2019) 74:175–87. doi: 10.1097/FJC.0000000000000717 31356555

[5] Agostini L Martinon F Burns K McDermott MF Hawkins PN Tschopp J. NALP3 forms an IL-1beta-processing inflammasome with increased activity in Muckle-Wells autoinflammatory disorder. Immunity. (2004) 20:319–25. doi: 10.1016/S1074-7613(04)00046-9 15030775

[6] de Vasconcelos NM Lamkanfi M. Recent insights on inflammasomes, gasdermin pores, and pyroptosis. Cold Spring Harbor Perspect Biol. (2020) 12(5):a036392. doi: 10.1101/cshperspect.a036392 PMC7197430 31570336

[7] Abderrazak A Syrovets T Couchie D El Hadri K Friguet B Simmet T. NLRP3 inflammasome: from a danger signal sensor to a regulatory node of oxidative stress and inflammatory diseases. Redox Biol. (2015) 4:296–307. doi: 10.1016/j.redox.2015.01.008 25625584 PMC4315937

[8] Muroi M Tanamoto K. Zinc- and oxidative property-dependent degradation of pro-caspase-1 and NLRP3 by ziram in mouse macrophages. Toxicol Lett. (2015) 235:199–205. doi: 10.1016/j.toxlet.2015.04.012 25929180

[9] Ohto U. Activation and regulation mechanisms of NOD-like receptors based on structural biology. Front Immunol. (2022) 13:953530. doi: 10.3389/fimmu.2022.953530 36189327 PMC9520476

[10] Sun HJ Ren XS Xiong XQ Chen YZ Zhao MX Wang JJ. NLRP3 inflammasome activation contributes to VSMC phenotypic transformation and proliferation in hypertension. Cell Death Dis. (2017) 8:e3074. doi: 10.1038/cddis.2017.470 28981106 PMC5680591

[11] Qi J Yu XJ Shi XL Gao HL Yi QY Tan H. NF-κB blockade in hypothalamic paraventricular nucleus inhibits high-salt-induced hypertension through NLRP3 and caspase-1. Cardiovasc Toxicol. (2016) 16:345–54. doi: 10.1007/s12012-015-9344-9 26438340

[12] Yu JW Wu J Zhang Z Datta P Ibrahimi I Taniguchi S. Cryopyrin and pyrin activate caspase-1, but not NF-kappaB, via ASC oligomerization. Cell Death differentiation. (2006) 13:236–49. doi: 10.1038/sj.cdd.4401734 16037825

[13] Fernandes-Alnemri T Wu J Yu JW Datta P Miller B Jankowski W. The pyroptosome: a supramolecular assembly of ASC dimers mediating inflammatory cell death via caspase-1 activation. Cell Death differentiation. (2007) 14:1590–604. doi: 10.1038/sj.cdd.4402194 PMC3345951 17599095

[14] Sborgi L Ravotti F Dandey VP Dick MS Mazur A Reckel S. Structure and assembly of the mouse ASC inflammasome by combined NMR spectroscopy and cryo-electron microscopy. Proc Natl Acad Sci United States America. (2015) 112:13237–42. doi: 10.1073/pnas.1507579112 PMC4629351 26464513

[15] Lu A Magupalli VG Ruan J Yin Q Atianand MK Vos MR. Unified polymerization mechanism for the assembly of ASC-dependent inflammasomes. Cell. (2014) 156:1193–206. doi: 10.1016/j.cell.2014.02.008 PMC4000066 24630722

[16] Thornberry NA Bull HG Calaycay JR Chapman KT Howard AD Kostura MJ. A novel heterodimeric cysteine protease is required for interleukin-1 beta processing in monocytes. Nature. (1992) 356:768–74. doi: 10.1038/356768a0 1574116

[17] Guo H Callaway JB Ting JP. Inflammasomes: mechanism of action, role in disease, and therapeutics. Nat Med. (2015) 21:677–87. doi: 10.1038/nm.3893 PMC4519035 26121197

[18] Bauernfeind FG Horvath G Stutz A Alnemri ES MacDonald K Speert D. Cutting edge: NF-kappaB activating pattern recognition and cytokine receptors license NLRP3 inflammasome activation by regulating NLRP3 expression. J Immunol (Baltimore Md.: 1950). (2009) 183:787–91. doi: 10.4049/jimmunol.0901363 PMC2824855 19570822

[19] Kodi T Sankhe R Gopinathan A Nandakumar K Kishore A. New insights on NLRP3 inflammasome: mechanisms of activation, inhibition, and epigenetic regulation. J neuroimmune pharmacology: Off J Soc NeuroImmune Pharmacol. (2024) 19:7. doi: 10.1007/s11481-024-10101-5 PMC10904444 38421496

[20] Pourcet B Zecchin M Ferri L Beauchamp J Sitaula S Billon C. Nuclear receptor subfamily 1 group D member 1 regulates circadian activity of NLRP3 inflammasome to reduce the severity of fulminant hepatitis in mice. Gastroenterology. (2018) 154:1449–1464.e20. doi: 10.1053/j.gastro.2017.12.019 29277561 PMC5892845

[21] O’Siorain JR Cox SL Payet C Nally FK He Y Drewinksi TT. Time-of-day control of mitochondria regulates NLRP3 inflammasome activation in macrophages. FASEB journal: Off Publ Fed Am Societies Exp Biol. (2024) 38:e70235. doi: 10.1096/fj.202400508RR PMC11669068 39686706

[22] Arioz BI Tarakcioglu E Olcum M Genc S. The role of melatonin on NLRP3 inflammasome activation in diseases. Antioxid (Basel Switzerland). (2021) 10(7):1020. doi: 10.3390/antiox10071020 PMC8300798 34202842

[23] Billon C Murray MH Avdagic A Burris TP. RORγ regulates the NLRP3 inflammasome. J Biol Chem. (2019) 294:10–9. doi: 10.1074/jbc.AC118.002127 PMC6322869 30455347

[24] Kou L Chi X Sun Y Han C Wan F Hu J. The circadian clock protein Rev-erbα provides neuroprotection and attenuates neuroinflammation against Parkinson’s disease via the microglial NLRP3 inflammasome. J Neuroinflamm. (2022) 19:133. doi: 10.1186/s12974-022-02494-y PMC9169406 35668454

[25] Guo DK Zhu Y Sun HY Xu XY Zhang S Hao ZB. Pharmacological activation of REV-ERBα represses LPS-induced microglial activation through the NF-κB pathway. Acta Pharmacol Sin. (2019) 40:26–34. doi: 10.1038/s41401-018-0064-0 29950615 PMC6318300

[26] Kawaguchi M Takahashi M Hata T Kashima Y Usui F Morimoto H. Inflammasome activation of cardiac fibroblasts is essential for myocardial ischemia/reperfusion injury. Circulation. (2011) 123:594–604. doi: 10.1161/CIRCULATIONAHA.110.982777 21282498

[27] Wang L Qu P Zhao J Chang Y. NLRP3 and downstream cytokine expression elevated in the monocytes of patients with coronary artery disease. Arch Med Sci: AMS. (2014) 10:791–800. doi: 10.5114/aoms.2014.44871 25276166 PMC4175781

[28] Toldo S Marchetti C Mauro AG Chojnacki J Mezzaroma E Carbone S. Inhibition of the NLRP3 inflammasome limits the inflammatory injury following myocardial ischemia-reperfusion in the mouse. Int J Cardiol. (2016) 209:215–20. doi: 10.1016/j.ijcard.2016.02.043 26896627

[29] Mastrocola R Penna C Tullio F Femminò S Nigro D Chiazza F. Pharmacological inhibition of NLRP3 inflammasome attenuates myocardial ischemia/reperfusion injury by activation of RISK and mitochondrial pathways. Oxid Med Cell Longevity. (2016) 2016:5271251. doi: 10.1155/2016/5271251 PMC5178375 28053692

[30] Zychlinsky A Prevost MC Sansonetti PJ. Shigella flexneri induces apoptosis in infected macrophages. Nature. (1992) 358:167–9. doi: 10.1038/358167a0 1614548

[31] Van Opdenbosch N Lamkanfi M. Caspases in cell death, inflammation, and disease. Immunity. (2019) 50:1352–64. doi: 10.1016/j.immuni.2019.05.020 PMC6611727 31216460

[32] Zhong X Zeng H Zhou Z Su Y Cheng H Hou Y. Structural mechanisms for regulation of GSDMB pore-forming activity. Nature. (2023) 616:598–605. doi: 10.1038/s41586-023-05872-5 36991125

[33] Zhou R Yazdi AS Menu P Tschopp J. A role for mitochondria in NLRP3 inflammasome activation. Nature. (2011) 469:221–5. doi: 10.1038/nature09663 21124315

[34] Andrei C Margiocco P Poggi A Lotti LV Torrisi MR Rubartelli A. and A2 control lysosome-mediated IL-1 beta secretion: Implications for inflammatory processes. Proc Natl Acad Sci United States America. (2004) 101:9745–50. doi: 10.1073/pnas.0308558101 PMC470745 15192144

[35] Piccini A Carta S Tassi S Lasiglié D Fossati G Rubartelli A. ATP is released by monocytes stimulated with pathogen-sensing receptor ligands and induces IL-1beta and IL-18 secretion in an autocrine way. Proc Natl Acad Sci United States America. (2008) 105:8067–72. doi: 10.1073/pnas.0709684105 PMC2430360 18523012

[36] Schmid-Burgk JL Gaidt MM Schmidt T Ebert TS Bartok E Hornung V. Caspase-4 mediates non-canonical activation of the NLRP3 inflammasome in human myeloid cells. Eur J Immunol. (2015) 45:2911–7. doi: 10.1002/eji.201545523 26174085

[37] Rühl S Broz P. Caspase-11 activates a canonical NLRP3 inflammasome by promoting K(+) efflux. Eur J Immunol. (2015) 45:2927–36. doi: 10.1002/eji.201545772 26173909

[38] Brough D Le Feuvre RA Wheeler RD Solovyova N Hilfiker S Rothwell NJ. Ca2+ stores and Ca2+ entry differentially contribute to the release of IL-1 beta and IL-1 alpha from murine macrophages. J Immunol (Baltimore Md.: 1950). (2003) 170:3029–36. doi: 10.4049/jimmunol.170.6.3029 12626557

[39] Murakami T Ockinger J Yu J Byles V McColl A Hofer AM. Critical role for calcium mobilization in activation of the NLRP3 inflammasome. Proc Natl Acad Sci United States America. (2012) 109:11282–7. doi: 10.1073/pnas.1117765109 PMC3396518 22733741

[40] Lee GS Subramanian N Kim AI Aksentijevich I Goldbach-Mansky R Sacks DB. The calcium-sensing receptor regulates the NLRP3 inflammasome through Ca2+ and cAMP. Nature. (2012) 492:123–7. doi: 10.1038/nature11588 PMC4175565 23143333

[41] Zhang X Hong S Qi S Liu W Zhang X Shi Z. NLRP3 inflammasome is involved in calcium-sensing receptor-induced aortic remodeling in SHRs. Mediators Inflammation. (2019) 2019:6847087. doi: 10.1155/2019/6847087 PMC6393924 30906225

[42] Liu W Sun J Guo Y Liu N Ding X Zhang X. Calhex231 ameliorates myocardial fibrosis post myocardial infarction in rats through the autophagy-NLRP3 inflammasome pathway in macrophages. J Cell Mol Med. (2020) 24:13440–53. doi: 10.1111/jcmm.v24.22 PMC7701583 33043596

[43] Martinon F Pétrilli V Mayor A Tardivel A Tschopp J. Gout-associated uric acid crystals activate the NALP3 inflammasome. Nature. (2006) 440:237–41. doi: 10.1038/nature04516 16407889

[44] Verhoef PA Kertesy SB Lundberg K Kahlenberg JM Dubyak GR. Inhibitory effects of chloride on the activation of caspase-1, IL-1beta secretion, and cytolysis by the P2X7 receptor. J Immunol (Baltimore Md.: 1950). (2005) 175:7623–34. doi: 10.4049/jimmunol.175.11.7623 16301672

[45] Groves DJ Batten TF. Direct control of the gonadotroph in a teleost, Poecilia latipinna: gonadal steroids. Gen Comp Endocrinol. (1986) 61:402–16. doi: 10.1016/0016-6480(86)90226-1 3956992

[46] Van Gele M Speleman F Vandesompele J Van Roy N Leonard JH. Characteristic pattern of chromosomal gains and losses in Merkel cell carcinoma detected by comparative genomic hybridization. Cancer Res. (1998) 58:1503–8. 9537255

[47] Domingo-Fernández R Coll RC Kearney J Breit S O’Neill LAJ. The intracellular chloride channel proteins CLIC1 and CLIC4 induce IL-1β transcription and activate the NLRP3 inflammasome. J Biol Chem. (2017) 292:12077–87. doi: 10.1074/jbc.M117.797126 PMC5519359 28576828

[48] Hornung V Bauernfeind F Halle A Samstad EO Kono H Rock KL. Silica crystals and aluminum salts activate the NALP3 inflammasome through phagosomal destabilization. Nat Immunol. (2008) 9:847–56. doi: 10.1038/ni.1631 PMC2834784 18604214

[49] Zhong Z Zhai Y Liang S Mori Y Han R Sutterwala FS. TRPM2 links oxidative stress to NLRP3 inflammasome activation. Nat Commun. (2013) 4:1611. doi: 10.1038/ncomms2608 23511475 PMC3605705

[50] Shao A Wu H Hong Y Tu S Sun X Wu Q. Hydrogen-rich saline attenuated subarachnoid hemorrhage-induced early brain injury in rats by suppressing inflammatory response: possible involvement of NF-κB pathway and NLRP3 inflammasome. Mol Neurobiol. (2016) 53:3462–76. doi: 10.1007/s12035-015-9242-y 26091790

[51] Nie Y Liu Q Zhang W Wan Y Huang C Zhu X. Ursolic acid reverses liver fibrosis by inhibiting NOX4/NLRP3 inflammasome pathways and bacterial dysbiosis. Gut Microbes. (2021) 13:1972746. doi: 10.1080/19490976.2021.1972746 34530693 PMC8451456

[52] Abais JM Xia M Zhang Y Boini KM Li PL. Redox regulation of NLRP3 inflammasomes: ROS as trigger or effector? Antioxid Redox Signaling. (2015) 22:1111–29. doi: 10.1080/19490976.2021.1972746 PMC4403231 25330206

[53] Nakahira K Haspel JA Rathinam VA Lee SJ Dolinay T Lam HC. Autophagy proteins regulate innate immune responses by inhibiting the release of mitochondrial DNA mediated by the NALP3 inflammasome. Nat Immunol. (2011) 12:222–30. doi: 10.1038/ni.1980 PMC3079381 21151103

[54] Shimada K Crother TR Karlin J Dagvadorj J Chiba N Chen S. Oxidized mitochondrial DNA activates the NLRP3 inflammasome during apoptosis. Immunity. (2012) 36:401–14. doi: 10.1016/j.immuni.2012.01.009 PMC3312986 22342844

[55] Zhong Z Liang S Sanchez-Lopez E He F Shalapour S Lin XJ. New mitochondrial DNA synthesis enables NLRP3 inflammasome activation. Nature. (2018) 560:198–203. doi: 10.1038/s41586-018-0372-z 30046112 PMC6329306

[56] Duewell P Kono H Rayner KJ Sirois CM Vladimer G Bauernfeind FG. NLRP3 inflammasomes are required for atherogenesis and activated by cholesterol crystals. Nature. (2010) 464:1357–61. doi: 10.1038/nature08938 PMC2946640 20428172

[57] Cassel SL Eisenbarth SC Iyer SS Sadler JJ Colegio OR Tephly LA. The Nalp3 inflammasome is essential for the development of silicosis. Proc Natl Acad Sci United States America. (2008) 105:9035–40. doi: 10.1073/pnas.0803933105 PMC2449360 18577586

[58] Ebrahimi T Rust M Kaiser SN Slowik A Beyer C Koczulla AR. [amp]]alpha;1-antitrypsin mitigates NLRP3-inflammasome activation in amyloid β(1-42)-stimulated murine astrocytes. J Neuroinflamm. (2018) 15:282. doi: 10.1186/s12974-018-1319-x PMC6158809 30261895

[59] Orlowski GM Colbert JD Sharma S Bogyo M Robertson SA Rock KL. Multiple cathepsins promote pro-IL-1β Synthesis and NLRP3-mediated IL-1β Activation. J Immunol (Baltimore Md.: 1950). (2015) 195:1685–97. doi: 10.4049/jimmunol.1500509 PMC4530060 26195813

[60] Hoseini Z Sepahvand F Rashidi B Sahebkar A Masoudifar A Mirzaei H. NLRP3 inflammasome: Its regulation and involvement in atherosclerosis. J Cell Physiol. (2018) 233:2116–32. doi: 10.1002/jcp.v233.3 28345767

[61] Gage J Hasu M Thabet M Whitman SC. Caspase-1 deficiency decreases atherosclerosis in apolipoprotein E-null mice. Can J Cardiol. (2012) 28:222–9. doi: 10.1016/j.cjca.2011.10.013 22265992

[62] Madaudo C Coppola G Parlati ALM Corrado E. Discovering inflammation in atherosclerosis: insights from pathogenic pathways to clinical practice. Int J Mol Sci. (2024) 25(11):6016. doi: 10.3390/ijms25116016 38892201 PMC11173271

[63] van der Heijden T Kritikou E Venema W van Duijn J van Santbrink PJ Slütter B. NLRP3 inflammasome inhibition by MCC950 reduces atherosclerotic lesion development in apolipoprotein E-deficient mice-brief report. Arteriosclerosis Thrombosis Vasc Biol. (2017) 37:1457–61. doi: 10.1161/ATVBAHA.117.309575 28596375

[64] Westerterp M Fotakis P Ouimet M Bochem AE Zhang H Molusky MM. Cholesterol efflux pathways suppress inflammasome activation, NETosis, and atherogenesis. Circulation. (2018) 138:898–912. doi: 10.1161/CIRCULATIONAHA.117.032636 29588315 PMC6160368

[65] Vedre A Pathak DR Crimp M Lum C Koochesfahani M Abela GS. Physical factors that trigger cholesterol crystallization leading to plaque rupture. Atherosclerosis. (2009) 203:89–96. doi: 10.1016/j.atherosclerosis.2008.06.027 18703195

[66] Thazhathveettil J Kumawat AK Demirel I Sirsjö A Paramel GV. Vascular smooth muscle cells in response to cholesterol crystals modulates inflammatory cytokines release and promotes neutrophil extracellular trap formation. Mol Med (Cambridge Mass). (2024) 30:42. doi: 10.1186/s10020-024-00809-8 38519881 PMC10960408

[67] Kalugotla G He L Weber KJ Daemen S Reller A Razani B. Frontline Science: Acyl-CoA synthetase 1 exacerbates lipotoxic inflammasome activation in primary macrophages. J Leukocyte Biol. (2019) 106:803–14. doi: 10.1002/JLB.3HI0219-045RR PMC7039344 31166619

[68] Sergin I Evans TD Zhang X Bhattacharya S Stokes CJ Song E. Exploiting macrophage autophagy-lysosomal biogenesis as a therapy for atherosclerosis. Nat Commun. (2017) 8:15750. doi: 10.1038/ncomms15750 28589926 PMC5467270

[69] Sergin I Bhattacharya S Emanuel R Esen E Stokes CJ Evans TD. Inclusion bodies enriched for p62 and polyubiquitinated proteins in macrophages protect against atherosclerosis. Sci Signaling. (2016) 9:ra2. doi: 10.1126/scisignal.aad5614 PMC5023144 26732762

[70] Niyonzima N Bakke SS Gregersen I Holm S Sandanger Ø Orrem HL. Cholesterol crystals use complement to increase NLRP3 signaling pathways in coronary and carotid atherosclerosis. EBioMedicine. (2020) 60:102985. doi: 10.1016/j.ebiom.2020.102985 32927275 PMC7494683

[71] Warnatsch A Ioannou M Wang Q Papayannopoulos V. Inflammation. Neutrophil extracellular traps license macrophages for cytokine production in atherosclerosis. Sci (New York N.Y.). (2015) 349:316–20. doi: 10.1126/science.aaa8064 PMC4854322 26185250

[72] Knight JS Luo W O’Dell AA Yalavarthi S Zhao W Subramanian V. Peptidylarginine deiminase inhibition reduces vascular damage and modulates innate immune responses in murine models of atherosclerosis. Circ Res. (2014) 114:947–56. doi: 10.1161/CIRCRESAHA.114.303312 PMC4185401 24425713

[73] McGraw KE Schilling K Glabonjat RA Galvez-Fernandez M Domingo-Relloso A Martinez-Morata I. Urinary metal levels and coronary artery calcification: longitudinal evidence in the multi-ethnic study of atherosclerosis. J Am Coll Cardiol. (2024) 84:1545–57. doi: 10.1016/j.jacc.2024.07.020 PMC11804863 39297845

[74] Wilkins JT Ning H Allen NB Zheutlin A Shah NS Feinstein MJ. Prediction of cumulative exposure to atherogenic lipids during early adulthood. J Am Coll Cardiol. (2024) 84:961–73. doi: 10.1016/j.jacc.2024.05.070 PMC13454923 39232632

[75] Nabel EG Braunwald E. A tale of coronary artery disease and myocardial infarction. New Engl J Med. (2012) 366:54–63. doi: 10.1056/NEJMra1112570 22216842

[76] Bochkov V Gesslbauer B Mauerhofer C Philippova M Erne P Oskolkova OV. Pleiotropic effects of oxidized phospholipids. Free Radical Biol Med. (2017) 111:6–24. doi: 10.1016/j.freeradbiomed.2016.12.034 28027924

[77] Binder CJ Papac-Milicevic N Witztum JL. Innate sensing of oxidation-specific epitopes in health and disease. Nat Rev Immunol. (2016) 16:485–97. doi: 10.1038/nri.2016.63 PMC7097710 27346802

[78] Seefeld U Bansky G Jaeger M Schmid M. Prevention of hepatitis B virus transmission by the gastrointestinal fibrescope. Successful disinfection with an aldehyde liquid. Endoscopy. (1981) 13:238–9. doi: 10.1055/s-2007-1021694 7297512

[79] Sheedy FJ Grebe A Rayner KJ Kalantari P Ramkhelawon B Carpenter SB. CD36 coordinates NLRP3 inflammasome activation by facilitating intracellular nucleation of soluble ligands into particulate ligands in sterile inflammation. Nat Immunol. (2013) 14:812–20. doi: 10.1038/ni.2639 PMC3720827 23812099

[80] Solini A Menini S Rossi C Ricci C Santini E Blasetti Fantauzzi C. The purinergic 2X7 receptor participates in renal inflammation and injury induced by high-fat diet: possible role of NLRP3 inflammasome activation. J Pathol. (2013) 231:342–53. doi: 10.1002/path.4237 23843215

[81] Stachon P Heidenreich A Merz J Hilgendorf I Wolf D Willecke F. P2X(7) deficiency blocks lesional inflammasome activity and ameliorates atherosclerosis in mice. Circulation. (2017) 135:2524–33. doi: 10.1161/CIRCULATIONAHA.117.027400 28377486

[82] Gomez D Baylis RA Durgin BG Newman AAC Alencar GF Mahan S. Interleukin-1β has atheroprotective effects in advanced atherosclerotic lesions of mice. Nat Med. (2018) 24:1418–29. doi: 10.1038/s41591-018-0124-5 PMC6130822 30038218

[83] Chiabrando JG Bonaventura A Vecchié A Wohlford GF Mauro AG Jordan JH. Management of acute and recurrent pericarditis: JACC state-of-the-art review. J Am Coll Cardiol. (2020) 75:76–92. doi: 10.1016/j.jacc.2019.11.021 31918837

[84] Vecchié A Chiabrando JG Dell MS Bonaventura A Mauro AG Wohlford G. Clinical presentation and outcomes of acute pericarditis in a large urban hospital in the United States of america. Chest. (2020) 158:2556–67. doi: 10.1016/j.chest.2020.07.039 PMC7768931 32717264

[85] Imazio M Bobbio M Cecchi E Demarie D Demichelis B Pomari F. Colchicine in addition to conventional therapy for acute pericarditis: results of the COlchicine for acute PEricarditis (COPE) trial. Circulation. (2005) 112:2012–6. doi: 10.1161/CIRCULATIONAHA.105.542738 16186437

[86] Lazarou E Tsioufis P Vlachopoulos C Tsioufis C Lazaros G. Acute pericarditis: update. Curr Cardiol Rep. (2022) 24:905–13. doi: 10.1007/s11886-022-01710-8 PMC9122084 35595949

[87] Renaudin F Orliaguet L Castelli F Fenaille F Prignon A Alzaid F. Gout and pseudo-gout-related crystals promote GLUT1-mediated glycolysis that governs NLRP3 and interleukin-1β activation on macrophages. Ann Rheumatic Dis. (2020) 79:1506–14. doi: 10.1136/annrheumdis-2020-217342 32699039

[88] Brucato A Imazio M Gattorno M Lazaros G Maestroni S Carraro M. Effect of anakinra on recurrent pericarditis among patients with colchicine resistance and corticosteroid dependence: the AIRTRIP randomized clinical trial. Jama. (2016) 316:1906–12. doi: 10.1001/jama.2016.15826 27825009

[89] Bonaventura A Thomas GK Golino M Mauro AG Vecchié A Del Buono MG. Novel pathophysiological, diagnostic and therapeutic concepts in acute and recurrent pericarditis. Rev Cardiovasc Med. (2023) 24:77. doi: 10.31083/j.rcm2403077 39077487 PMC11264016

[90] Vecchié A Bonaventura A Golino M Thomas G Abbate A. Novel therapeutic insights into the treatment of pericarditis: targeting the innate immune system. J Cardiovasc Pharmacol. (2024) 83:377–83. doi: 10.1097/FJC.0000000000001553 38422218

[91] Mauro AG Bonaventura A Vecchié A Mezzaroma E Carbone S Narayan P. The role of NLRP3 inflammasome in pericarditis: potential for therapeutic approaches. JACC Basic To Trans Sci. (2021) 6:137–50. doi: 10.1016/j.jacbts.2020.11.016 PMC7907621 33665514

[92] Vecchié A Del Buono MG Chiabrando GJ Dentali F Abbate A Bonaventura A. Interleukin-1 and the NLRP3 inflammasome in pericardial disease. Curr Cardiol Rep. (2021) 23:157. doi: 10.1007/s11886-021-01589-x 34599390 PMC8485973

[93] Vukadinović D Lauder L Kandzari DE Bhatt DL Kirtane AJ Edelman ER. Effects of catheter-based renal denervation in hypertension: A systematic review and meta-analysis. Circulation. (2024) 150:1599–611. doi: 10.1161/CIRCULATIONAHA.124.069709 PMC11560572 39355923

[94] Damaskos C Litos A Dimitroulis D Antoniou EA Mantas D Kontzoglou K. Cardiovascular effects of metabolic surgery on type 2 diabetes. Curr Cardiol Rev. (2020) 16:275–84. doi: 10.2174/1573403X16666200220120226 PMC7903510 32077829

[95] Ye J Ji Q Liu J Liu L Huang Y Shi Y. Interleukin 22 promotes blood pressure elevation and endothelial dysfunction in angiotensin II-treated mice. J Am Heart Assoc. (2017) 6(10):e005875. doi: 10.1161/JAHA.117.005875 28974499 PMC5721831

[96] Shirasuna K Karasawa T Takahashi M. Role of the NLRP3 inflammasome in preeclampsia. Front Endocrinol. (2020) 11:80. doi: 10.3389/fendo.2020.00080 PMC7053284 32161574

[97] Shirasuna K Karasawa T Usui F Kobayashi M Komada T Kimura H. NLRP3 deficiency improves angiotensin II-induced hypertension but not fetal growth restriction during pregnancy. Endocrinology. (2015) 156:4281–92. doi: 10.1210/en.2015-1408 26360504

[98] Bjornstad P Laffel L Lynch J El Ghormli L Weinstock RS Tollefsen SE. Elevated serum uric acid is associated with greater risk for hypertension and diabetic kidney diseases in obese adolescents with type 2 diabetes: an observational analysis from the treatment options for type 2 diabetes in adolescents and youth (TODAY) study. Diabetes Care. (2019) 42:1120–8. doi: 10.2337/dc18-2147 PMC6609951 30967435

[99] Su Q Liu JJ Cui W Shi XL Guo J Li HB. Alpha lipoic acid supplementation attenuates reactive oxygen species in hypothalamic paraventricular nucleus and sympathoexcitation in high salt-induced hypertension. Toxicol Lett. (2016) 241:152–8. doi: 10.1016/j.toxlet.2015.10.019 26518973

[100] Gan W Li T Ren J Li C Liu Z Yang M. Serum-glucocorticoid-regulated kinase 1 contributes to mechanical stretch-induced inflammatory responses in cardiac fibroblasts. Mol Cell Biochem. (2018) 445:67–78. doi: 10.1007/s11010-017-3252-1 29243066

[101] Gan W Ren J Li T Lv S Li C Liu Z. The SGK1 inhibitor EMD638683, prevents Angiotensin II-induced cardiac inflammation and fibrosis by blocking NLRP3 inflammasome activation. Biochim Biophys Acta Mol Basis Dis. (2018) 1864:1–10. doi: 10.1016/j.bbadis.2017.10.001 28986310

[102] Liu Y Yin HL Li C Jiang F Zhang SJ Zhang XR. Sinapine thiocyanate ameliorates vascular endothelial dysfunction in hypertension by inhibiting activation of the NLRP3 inflammasome. Front Pharmacol. (2020) 11:620159. doi: 10.3389/fphar.2020.620159 33633569 PMC7901921

[103] Ge R Chen JL Zheng F Yin SM Dai M Wang YM. Asprosin promotes vascular inflammation via TLR4-NFκB-mediated NLRP3 inflammasome activation in hypertension. Heliyon. (2024) 10:e31659. doi: 10.1016/j.heliyon.2024.e31659 38841464 PMC11152944

[104] Soden PA Zettervall SL Ultee KH Darling JD Buck DB Hile CN. Outcomes for symptomatic abdominal aortic aneurysms in the American College of Surgeons National Surgical Quality Improvement Program. J Vasc Surg. (2016) 64:297–305. doi: 10.1016/j.jvs.2016.02.055 27146791 PMC5065370

[105] Dale MA Ruhlman MK Baxter BT. Inflammatory cell phenotypes in AAAs: their role and potential as targets for therapy. Arteriosclerosis Thrombosis Vasc Biol. (2015) 35:1746–55. doi: 10.1161/ATVBAHA.115.305269 PMC4514552 26044582

[106] Wang J Ye W Zou J Yang P Jin M Zheng Z. Targeting the smooth muscle cell Keap1-Nrf2-GSDMD-pyroptosis axis by cryptotanshinone prevents abdominal aortic aneurysm formation. Theranostics. (2024) 14:6516–42. doi: 10.7150/thno.98400 PMC11519792 39479449

[107] Pi S Xiong S Yuan Y Deng H. The role of inflammasome in abdominal aortic aneurysm and its potential drugs. Int J Mol Sci. (2024) 25(9):5001. doi: 10.3390/ijms25095001 38732221 PMC11084561

[108] Ramprasath T Han YM Zhang D Yu CJ Zou MH. Tryptophan catabolism and inflammation: A novel therapeutic target for aortic diseases. Front Immunol. (2021) 12:731701. doi: 10.3389/fimmu.2021.731701 34630411 PMC8496902

[109] Ren P Wu D Appel R Zhang L Zhang C Luo W. Targeting the NLRP3 inflammasome with inhibitor MCC950 prevents aortic aneurysms and dissections in mice. J Am Heart Assoc. (2020) 9:e014044. doi: 10.1161/JAHA.119.014044 32223388 PMC7428617

[110] Monin A Didier R Leclercq T Chagué F Rochette L Danchin N. Coronary artery embolism and acute coronary syndrome: A critical appraisal of existing data. Trends Cardiovasc Med. (2024) 34:50–6. doi: 10.1016/j.tcm.2022.07.004 35868593

[111] Payne FM Dabb AR Harrison JC Sammut IA. Inhibitors of NLRP3 inflammasome formation: A cardioprotective role for the gasotransmitters carbon monoxide, nitric oxide, and hydrogen sulphide in acute myocardial infarction. Int J Mol Sci. (2024) 25(17):9247. doi: 10.3390/ijms25179247 39273196 PMC11395567

[112] Mezzaroma E Toldo S Farkas D Seropian IM Van Tassell BW Salloum FN. The inflammasome promotes adverse cardiac remodeling following acute myocardial infarction in the mouse. Proc Natl Acad Sci United States America. (2011) 108:19725–30. doi: 10.1073/pnas.1108586108 PMC3241791 22106299

[113] Marchetti C Toldo S Chojnacki J Mezzaroma E Liu K Salloum FN. Pharmacologic inhibition of the NLRP3 inflammasome preserves cardiac function after ischemic and nonischemic injury in the mouse. J Cardiovasc Pharmacol. (2015) 66:1–8. doi: 10.1097/FJC.0000000000000247 25915511 PMC4500673

[114] Abbate A Salloum FN Vecile E Das A Hoke NN Straino S. Anakinra, a recombinant human interleukin-1 receptor antagonist, inhibits apoptosis in experimental acute myocardial infarction. Circulation. (2008) 117:2670–83. doi: 10.1161/CIRCULATIONAHA.107.740233 18474815

[115] Lugrin J Parapanov R Rosenblatt-Velin N Rignault-Clerc S Feihl F Waeber B. Cutting edge: IL-1α is a crucial danger signal triggering acute myocardial inflammation during myocardial infarction. J Immunol (Baltimore Md.: 1950). (2015) 194:499–503. doi: 10.4049/jimmunol.1401948 PMC4278196 25505286

[116] Maron BJ Towbin JA Thiene G Antzelevitch C Corrado D Arnett D. Contemporary definitions and classification of the cardiomyopathies: an American Heart Association Scientific Statement from the Council on Clinical Cardiology, Heart Failure and Transplantation Committee; Quality of Care and Outcomes Research and Functional Genomics and Translational Biology Interdisciplinary Working Groups; and Council on Epidemiology and Prevention. Circulation. (2006) 113:1807–16. doi: 10.1161/CIRCULATIONAHA.106.174287 16567565

[117] Sikking MA Stroeks S Henkens M Raafs AG Cossins B van Deuren RC. Clonal hematopoiesis has prognostic value in dilated cardiomyopathy independent of age and clone size. JACC. Heart Failure. (2024) 12:905–14. doi: 10.1016/j.jchf.2023.06.037 37638520

[118] Zeng C Duan F Hu J Luo B Huang B Lou X. NLRP3 inflammasome-mediated pyroptosis contributes to the pathogenesis of non-ischemic dilated cardiomyopathy. Redox Biol. (2020) 34:101523. doi: 10.1016/j.redox.2020.101523 32273259 PMC7327979

[119] Zhang L Ai C Bai M Niu J Zhang Z. NLRP3 inflammasome/pyroptosis: A key driving force in diabetic cardiomyopathy. Int J Mol Sci. (2022) 23(18):10632. doi: 10.3390/ijms231810632 36142531 PMC9501057

[120] Zhang W Tao A Lan T Cepinskas G Kao R Martin CM. Carbon monoxide releasing molecule-3 improves myocardial function in mice with sepsis by inhibiting NLRP3 inflammasome activation in cardiac fibroblasts. Basic Res Cardiol. (2017) 112:16. doi: 10.1007/s00395-017-0603-8 28168403

[121] Saeedi P Salpea P Karuranga S Petersohn I Malanda B Gregg EW. Mortality attributable to diabetes in 20-79 years old adults, 2019 estimates: Results from the International Diabetes Federation Diabetes Atlas, 9(th) edition. Diabetes Res Clin Pract. (2020) 162:108086. doi: 10.1016/j.diabres.2020.108086 32068099

[122] Mizamtsidi M Paschou SA Grapsa J Vryonidou A. Diabetic cardiomyopathy: a clinical entity or a cluster of molecular heart changes? Eur J Clin Invest. (2016) 46:947–53. doi: 10.1111/eci.12673 27600276

[123] Kim SJ Lee SM. NLRP3 inflammasome activation in D-galactosamine and lipopolysaccharide-induced acute liver failure: role of heme oxygenase-1. Free Radical Biol Med. (2013) 65:997–1004. doi: 10.1016/j.freeradbiomed.2013.08.178 23994575

[124] Feng H Gu J Gou F Huang W Gao C Chen G. High glucose and lipopolysaccharide prime NLRP3 inflammasome via ROS/TXNIP pathway in mesangial cells. J Diabetes Res. (2016) 2016:6973175. doi: 10.1155/2016/6973175 26881256 PMC4736396

[125] Han Y Xu X Tang C Gao P Chen X Xiong X. Reactive oxygen species promote tubular injury in diabetic nephropathy: The role of the mitochondrial ros-txnip-nlrp3 biological axis. Redox Biol. (2018) 16:32–46. doi: 10.1016/j.redox.2018.02.013 29475133 PMC5842313

[126] Chen W Zhao M Zhao S Lu Q Ni L Zou C. Activation of the TXNIP/NLRP3 inflammasome pathway contributes to inflammation in diabetic retinopathy: a novel inhibitory effect of minocycline. Inflammation Res. (2017) 66:157–66. doi: 10.1007/s00011-016-1002-6 27785530

[127] Tosswill JH Ades AE Peckham C Mortimer PP Weber JN. Infection with human T cell leukaemia/lymphoma virus type I in patients attending an antenatal clinic in London. BMJ (Clin Res ed.). (1990) 301:95–6. doi: 10.1136/bmj.301.6743.95 PMC1663411 2390591

[128] Bryant C Fitzgerald KA. Molecular mechanisms involved in inflammasome activation. Trends Cell Biol. (2009) 19:455–64. doi: 10.1016/j.tcb.2009.06.002 19716304

[129] Lv S Liu H Wang H. The interplay between autophagy and NLRP3 inflammasome in ischemia/reperfusion injury. Int J Mol Sci. (2021) 22(16):8773. doi: 10.3390/ijms22168773 34445481 PMC8395601

[130] Zhou R Tardivel A Thorens B Choi I Tschopp J. Thioredoxin-interacting protein links oxidative stress to inflammasome activation. Nat Immunol. (2010) 11:136–40. doi: 10.1038/ni.1831 20023662

[131] Kolodkin-Gal I Parsek MR Patrauchan MA. The roles of calcium signaling and calcium deposition in microbial multicellularity. Trends Microbiol. (2023) 31:1225–37. doi: 10.1016/j.tim.2023.06.005 PMC10772221 37429751

[132] Erciyes D Bora ES Tekindal MA Erbaş O. Demonstration of the protective effect of vinpocetine in diabetic cardiomyopathy. J Clin Med. (2024) 13(16):4637. doi: 10.3390/jcm13164637 39200779 PMC11354616

[133] Pasqualetti G Seghieri M Santini E Rossi C Vitolo E Giannini L. P2X(7) receptor and APOE polymorphisms and survival from heart failure: A prospective study in frail patients in a geriatric unit. Aging Dis. (2017) 8:434–41. doi: 10.14336/AD.2016.1202 PMC5524806 28840058

[134] Vlachakis PK Theofilis P Kachrimanidis I Giannakopoulos K Drakopoulou M Apostolos A. The role of inflammasomes in heart failure. Int J Mol Sci. (2024) 25(10):5372. doi: 10.3390/ijms25105372 38791409 PMC11121241

[135] Roy R Wilcox J Webb AJ O’Gallagher K. Dysfunctional and dysregulated nitric oxide synthases in cardiovascular disease: mechanisms and therapeutic potential. Int J Mol Sci. (2023) 24(20):15200. doi: 10.3390/ijms242015200 37894881 PMC10607291

[136] Finkel MS Oddis CV Jacob TD Watkins SC Hattler BG Simmons RL. Negative inotropic effects of cytokines on the heart mediated by nitric oxide. Sci (New York N.Y.). (1992) 257:387–9. doi: 10.1126/science.1631560 1631560

[137] Van Tassell BW Arena RA Toldo S Mezzaroma E Azam T Seropian IM. Enhanced interleukin-1 activity contributes to exercise intolerance in patients with systolic heart failure. PloS One. (2012) 7:e33438. doi: 10.1371/journal.pone.0033438 22438931 PMC3306393

[138] Toldo S Abbate A. The role of the NLRP3 inflammasome and pyroptosis in cardiovascular diseases. Nat Rev Cardiol. (2024) 21:219–37. doi: 10.1038/s41569-023-00946-3 PMC11550901 37923829

[139] Dinarello CA Simon A van der Meer JW. Treating inflammation by blocking interleukin-1 in a broad spectrum of diseases. Nat Rev Drug Discovery. (2012) 11:633–52. doi: 10.1038/nrd3800 PMC3644509 22850787

[140] Leon MN Harrell LC Simosa HF Mahdi NA Pathan A Lopez-Cuellar J. Mitral balloon valvotomy for patients with mitral stenosis in atrial fibrillation: immediate and long-term results. J Am Coll Cardiol. (1999) 34:1145–52. doi: 10.1016/S0735-1097(99)00310-1 10520804

[141] Chung ES Packer M Lo KH Fasanmade AA Willerson JT. Randomized, double-blind, placebo-controlled, pilot trial of infliximab, a chimeric monoclonal antibody to tumor necrosis factor-alpha, in patients with moderate-to-severe heart failure: results of the anti-TNF Therapy Against Congestive Heart Failure (ATTACH) trial. Circulation. (2003) 107:3133–40. doi: 10.1161/01.CIR.0000077913.60364.D2 12796126

[142] Burkard T Pfister O Rickli H Follath F Hack D Zaker R. Prognostic impact of systemic inflammatory diseases in elderly patients with congestive heart failure. QJM: Monthly J Assoc Physicians. (2014) 107:131–8. doi: 10.1093/qjmed/hct205 24131549

[143] Bozkurt B Torre-Amione G Warren MS Whitmore J Soran OZ Feldman AM. Results of targeted anti-tumor necrosis factor therapy with etanercept (ENBREL) in patients with advanced heart failure. Circulation. (2001) 103:1044–7. doi: 10.1161/01.CIR.103.8.1044 11222463

[144] Hardy SA Liesinger L Patrick R Poettler M Rech L Gindlhuber J. Extracellular matrix protein-1 as a mediator of inflammation-induced fibrosis after myocardial infarction. JACC. Basic To Trans Sci. (2023) 8:1539–54. doi: 10.1016/j.jacbts.2023.05.010 PMC10774582 38205347

[145] Bracey NA Gershkovich B Chun J Vilaysane A Meijndert HC Wright JR Jr.. Mitochondrial NLRP3 protein induces reactive oxygen species to promote Smad protein signaling and fibrosis independent from the inflammasome. J Biol Chem. (2014) 289:19571–84. doi: 10.1074/jbc.M114.550624 PMC4094069 24841199

[146] Généreux P Sharma RP Cubeddu RJ Aaron L Abdelfattah OM Koulogiannis KP. The mortality burden of untreated aortic stenosis. J Am Coll Cardiol. (2023) 82:2101–9. doi: 10.1016/j.jacc.2023.09.796 37877909

[147] Huang Y Wang C Zhou T Xie F Liu Z Xu H. Lumican promotes calcific aortic valve disease through H3 histone lactylation. Eur Heart J. (2024) 45:3871–85. doi: 10.1093/eurheartj/ehae407 38976370

[148] Rutkovskiy A Malashicheva A Sullivan G Bogdanova M Kostareva A Stensløkken KO. Valve interstitial cells: the key to understanding the pathophysiology of heart valve calcification. J Am Heart Assoc. (2017) 6(9):e006339. doi: 10.1161/JAHA.117.006339 28912209 PMC5634284

[149] Cho KI Sakuma I Sohn IS Jo SH Koh KK. Inflammatory and metabolic mechanisms underlying the calcific aortic valve disease. Atherosclerosis. (2018) 277:60–5. doi: 10.1016/j.atherosclerosis.2018.08.029 30173080

[150] Rogers MA Bartoli-Leonard F Zheng KH Small AM Chen HY Clift CL. Major facilitator superfamily domain containing 5 inhibition reduces lipoprotein(a) uptake and calcification in valvular heart disease. Circulation. (2024) 149:391–401. doi: 10.1161/CIRCULATIONAHA.123.066822 37937463 PMC10842618

[151] Liu Z Liu Y Yu Z Tan C Pek N O’Donnell A. APOE-NOTCH axis governs elastogenesis during human cardiac valve remodeling. Nat Cardiovasc Res. (2024) 3:933–50. doi: 10.1038/s44161-024-00510-3 39196035

[152] Bhattacharyya S Tamaki Z Wang W Hinchcliff M Hoover P Getsios S. FibronectinEDA promotes chronic cutaneous fibrosis through Toll-like receptor signaling. Sci Trans Med. (2014) 6:232ra50. doi: 10.1126/scitranslmed.3008264 PMC4414050 24739758

[153] Dweck MR Khaw HJ Sng GK Luo EL Baird A Williams MC. Aortic stenosis, atherosclerosis, and skeletal bone: is there a common link with calcification and inflammation? Eur Heart J. (2013) 34:1567–74. doi: 10.1093/eurheartj/eht034 23391586

[154] Otto CM Kuusisto J Reichenbach DD Gown AM O’Brien KD. Characterization of the early lesion of ‘degenerative’ valvular aortic stenosis. Histological and immunohistochemical studies. Circulation. (1994) 90:844–53. doi: 10.1161/01.CIR.90.2.844 7519131

[155] Liu M Li F Huang Y Zhou T Chen S Li G. Caffeic acid phenethyl ester ameliorates calcification by inhibiting activation of the AKT/NF-κB/NLRP3 inflammasome pathway in human aortic valve interstitial cells. Front Pharmacol. (2020) 11:826. doi: 10.3389/fphar.2020.00826 32733235 PMC7358518

[156] Lu J Meng J Wu G Wei W Xie H Liu Y. Th1 cells reduce the osteoblast-like phenotype in valvular interstitial cells by inhibiting NLRP3 inflammasome activation in macrophages. Mol Med (Cambridge Mass). (2024) 30:110. doi: 10.1186/s10020-024-00882-z 39080527 PMC11287975

[157] Lu J Xie S Deng Y Xie X Liu Y. Blocking the NLRP3 inflammasome reduces osteogenic calcification and M1 macrophage polarization in a mouse model of calcified aortic valve stenosis. Atherosclerosis. (2022) 347:28–38. doi: 10.1016/j.atherosclerosis.2022.03.005 35299058

[158] Wu J Omene C Karkoszka J Bosland M Eckard J Klein CB. Caffeic acid phenethyl ester (CAPE), derived from a honeybee product propolis, exhibits a diversity of anti-tumor effects in pre-clinical models of human breast cancer. Cancer Lett. (2011) 308:43–53. doi: 10.1016/j.canlet.2011.04.012 21570765 PMC3144783

[159] Zhou J Zhu J Jiang L Zhang B Zhu D Wu Y. Interleukin 18 promotes myofibroblast activation of valvular interstitial cells. Int J Cardiol. (2016) 221:998–1003. doi: 10.1016/j.ijcard.2016.07.036 27441481

[160] Sasaki K Watanabe Y Kozuma K Kataoka A Hioki H Nagura F. Comparison of long-term mortality in patients who underwent transcatheter aortic valve replacement with or without anti-atherosclerotic therapy. Heart Vessels. (2021) 36:1892–902. doi: 10.1007/s00380-021-01873-4 34101028

[161] Long W Light PE Simpson SH. Glyburide use is associated with a greater likelihood of mortality or rehospitalization after acute coronary syndrome compared to gliclazide use in adults with type 2 diabetes: A cohort study. Diabetes Obes Metab. (2024) 26:5408–19. doi: 10.1111/dom.v26.11 39248222

[162] Lamkanfi M Mueller JL Vitari AC Misaghi S Fedorova A Deshayes K. Glyburide inhibits the Cryopyrin/Nalp3 inflammasome. J Cell Biol. (2009) 187:61–70. doi: 10.1083/jcb.200903124 19805629 PMC2762099

[163] Marchetti C Chojnacki J Toldo S Mezzaroma E Tranchida N Rose SW. A novel pharmacologic inhibitor of the NLRP3 inflammasome limits myocardial injury after ischemia-reperfusion in the mouse. J Cardiovasc Pharmacol. (2014) 63:316–22. doi: 10.1097/FJC.0000000000000053 PMC3980088 24336017

[164] Xu Y Xu Y Blevins H Guo C Biby S Wang XY. Development of sulfonamide-based NLRP3 inhibitors: Further modifications and optimization through structure-activity relationship studies. Eur J Medicinal Chem. (2022) 238:114468. doi: 10.1016/j.ejmech.2022.114468 PMC10084479 35635948

[165] Quader M Mezzaroma E Kenning K Toldo S. Targeting the NLRP3 inflammasome to reduce warm ischemic injury in donation after circulatory death heart. Clin Transplant. (2020) 34:e14044. doi: 10.1111/ctr.v34.10 32654189

[166] Carbone S Mauro AG Prestamburgo A Halquist MS Narayan P Potere N. An orally available NLRP3 inflammasome inhibitor prevents western diet-induced cardiac dysfunction in mice. J Cardiovasc Pharmacol. (2018) 72:303–7. doi: 10.1097/FJC.0000000000000628 PMC6511457 30422890

[167] Perregaux DG McNiff P Laliberte R Hawryluk N Peurano H Stam E. Identification and characterization of a novel class of interleukin-1 post-translational processing inhibitors. J Pharmacol Exp Ther. (2001) 299:187–97. doi: 10.1016/S0022-3565(24)29317-4 11561079

[168] Zhang X Xu A Lv J Zhang Q Ran Y Wei C. Development of small molecule inhibitors targeting NLRP3 inflammasome pathway for inflammatory diseases. Eur J Medicinal Chem. (2020) 185:111822. doi: 10.1016/j.ejmech.2019.111822 31699536

[169] Coll RC Hill JR Day CJ Zamoshnikova A Boucher D Massey NL. MCC950 directly targets the NLRP3 ATP-hydrolysis motif for inflammasome inhibition. Nat Chem Biol. (2019) 15:556–9. doi: 10.1038/s41589-019-0277-7 31086327

[170] Vande Walle L Stowe IB Šácha P Lee BL Demon D Fossoul A. MCC950/CRID3 potently targets the NACHT domain of wild-type NLRP3 but not disease-associated mutants for inflammasome inhibition. PloS Biol. (2019) 17:e3000354. doi: 10.1371/journal.pbio.3000529 31525186 PMC6762198

[171] van Hout GP Bosch L Ellenbroek GH de Haan JJ van Solinge WW Cooper MA. The selective NLRP3-inflammasome inhibitor MCC950 reduces infarct size and preserves cardiac function in a pig model of myocardial infarction. Eur Heart J. (2017) 38:828–36. doi: 10.1093/eurheartj/ehw247 27432019

[172] Gao R Shi H Chang S Gao Y Li X Lv C. The selective NLRP3-inflammasome inhibitor MCC950 reduces myocardial fibrosis and improves cardiac remodeling in a mouse model of myocardial infarction. Int Immunopharmacol. (2019) 74:105575. doi: 10.1016/j.intimp.2019.04.022 31299609

[173] Cheng P Yang G Zhao X Zeng W Sun D Zeng L. Precisely and efficiently enzyme response microspheres with immune removal escape loaded with MCC950 ameliorate cardiac dysfunction in acute myocardial infarction. J Biomed Nanotechnol. (2020) 16:153–65. doi: 10.1166/jbn.2020.2885 32252877

[174] Willeford A Suetomi T Nickle A Hoffman HM Miyamoto S Heller Brown J. CaMKIIδ-mediated inflammatory gene expression and inflammasome activation in cardiomyocytes initiate inflammation and induce fibrosis. JCI Insight. (2018) 3(12):e97054. doi: 10.1172/jci.insight.97054 29925681 PMC6124412

[175] Wang H Sun X Hodge HS Ferrario CM Groban L. NLRP3 inhibition improves heart function in GPER knockout mice. Biochem Biophys Res Commun. (2019) 514:998–1003. doi: 10.1016/j.bbrc.2019.05.045 31092335 PMC6545146

[176] Yao C Veleva T Scott L Jr. Cao S Li L Chen G. Enhanced cardiomyocyte NLRP3 inflammasome signaling promotes atrial fibrillation. Circulation. (2018) 138:2227–42. doi: 10.1161/CIRCULATIONAHA.118.035202 PMC6252285 29802206

[177] Juliana C Fernandes-Alnemri T Wu J Datta P Solorzano L Yu JW. Anti-inflammatory compounds parthenolide and Bay 11-7082 are direct inhibitors of the inflammasome. J Biol Chem. (2010) 285:9792–802. doi: 10.1074/jbc.M109.082305 PMC2843228 20093358

[178] Liu Y Lian K Zhang L Wang R Yi F Gao C. TXNIP mediates NLRP3 inflammasome activation in cardiac microvascular endothelial cells as a novel mechanism in myocardial ischemia/reperfusion injury. Basic Res Cardiol. (2014) 109:415. doi: 10.1007/s00395-014-0415-z 25015733

[179] Kim YS Kim JS Kwon JS Jeong MH Cho JG Park JC. BAY 11-7082, a nuclear factor-κB inhibitor, reduces inflammation and apoptosis in a rat cardiac ischemia-reperfusion injury model. Int Heart J. (2010) 51:348–53. doi: 10.1536/ihj.51.348 20966608

[180] Marchetti C Swartzwelter B Gamboni F Neff CP Richter K Azam T. OLT1177, a β-sulfonyl nitrile compound, safe in humans, inhibits the NLRP3 inflammasome and reverses the metabolic cost of inflammation. Proc Natl Acad Sci United States America. (2018) 115:E1530–e1539. doi: 10.1073/pnas.1716095115 PMC5816172 29378952

[181] Qiu Z Lei S Zhao B Wu Y Su W Liu M. NLRP3 inflammasome activation-mediated pyroptosis aggravates myocardial ischemia/reperfusion injury in diabetic rats. Oxid Med Cell Longevity. (2017) 2017:9743280. doi: 10.1155/2017/9743280 PMC5618779 29062465

[182] Lonnemann N Hosseini S Marchetti C Skouras DB Stefanoni D D’Alessandro A. The NLRP3 inflammasome inhibitor OLT1177 rescues cognitive impairment in a mouse model of Alzheimer’s disease. Proc Natl Acad Sci United States America. (2020) 117:32145–54. doi: 10.1073/pnas.2009680117 PMC7749353 33257576

[183] Sánchez-Fernández A Skouras DB Dinarello CA López-Vales R. OLT1177 (Dapansutrile), a selective NLRP3 inflammasome inhibitor, ameliorates experimental autoimmune encephalomyelitis pathogenesis. Front Immunol. (2019) 10:2578. doi: 10.3389/fimmu.2019.02578 31736980 PMC6839275

[184] Toldo S Mauro AG Cutter Z Van Tassell BW Mezzaroma E Del Buono MG. The NLRP3 inflammasome inhibitor, OLT1177 (Dapansutrile), reduces infarct size and preserves contractile function after ischemia reperfusion injury in the mouse. J Cardiovasc Pharmacol. (2019) 73:215–22. doi: 10.1097/FJC.0000000000000658 30747785

[185] Klück V Jansen T Janssen M Comarniceanu A Efdé M Tengesdal IW. Dapansutrile, an oral selective NLRP3 inflammasome inhibitor, for treatment of gout flares: an open-label, dose-adaptive, proof-of-concept, phase 2a trial. The Lancet. Rheumatology. (2020) 2:e270–80. doi: 10.1016/S2665-9913(20)30065-5 PMC7523621 33005902

[186] Wohlford GF Van Tassell BW Billingsley HE Kadariya D Canada JM Carbone S. Phase 1B, randomized, double-blinded, dose escalation, single-center, repeat dose safety and pharmacodynamics study of the oral NLRP3 inhibitor dapansutrile in subjects with NYHA II-III systolic heart failure. J Cardiovasc Pharmacol. (2020) 77:49–60. doi: 10.1097/FJC.0000000000000931 33235030 PMC7774821

[187] Cocco M Garella D Di Stilo A Borretto E Stevanato L Giorgis M. Electrophilic warhead-based design of compounds preventing NLRP3 inflammasome-dependent pyroptosis. J Medicinal Chem. (2014) 57:10366–82. doi: 10.1021/jm501072b 25418070

[188] Cocco M Miglio G Giorgis M Garella D Marini E Costale A. Design, synthesis, and evaluation of acrylamide derivatives as direct NLRP3 inflammasome inhibitors. ChemMedChem. (2016) 11:1790–803. doi: 10.1002/cmdc.201600055 26990578

[189] Darakhshan S Pour AB. Tranilast: a review of its therapeutic applications. Pharmacol Res. (2015) 91:15–28. doi: 10.1016/j.phrs.2014.10.009 25447595

[190] Huang Y Jiang H Chen Y Wang X Yang Y Tao J. Tranilast directly targets NLRP3 to treat inflammasome-driven diseases. EMBO Mol Med. (2018) 10(4):e8689. doi: 10.15252/emmm.201708689 29531021 PMC5887903

[191] Chen S Wang Y Pan Y Liu Y Zheng S Ding K. Novel role for tranilast in regulating NLRP3 ubiquitination, vascular inflammation, and atherosclerosis. J Am Heart Assoc. (2020) 9:e015513. doi: 10.1161/JAHA.119.015513 32476536 PMC7429049

[192] Umemura K Kikuchi S Suzuki Y Nakashima M. Inhibitory effect of tranilast on hypertrophic collagen production in the spontaneously hypertensive rat heart. Japanese J Pharmacol. (1998) 78:161–7. doi: 10.1254/jjp.78.161 9829619

[193] Lin HB Wei GS Li FX Guo WJ Hong P Weng YQ. Macrophage-NLRP3 inflammasome activation exacerbates cardiac dysfunction after ischemic stroke in a mouse model of diabetes. Neurosci Bull. (2020) 36:1035–45. doi: 10.1007/s12264-020-00544-0 PMC7475163 32683554

[194] Imazio M Brucato A Cemin R Ferrua S Belli R Maestroni S. Colchicine for recurrent pericarditis (CORP): a randomized trial. Ann Internal Med. (2011) 155:409–14. doi: 10.7326/0003-4819-155-7-201110040-00359 21873705

[195] Ridker PM. Anticytokine agents: targeting interleukin signaling pathways for the treatment of atherothrombosis. Circ Res. (2019) 124:437–50. doi: 10.1161/CIRCRESAHA.118.313129 PMC6386195 30702995

[196] Wohlford GF Buckley LF Vecchié A Kadariya D Markley R Trankle CR. Acute effects of interleukin-1 blockade using anakinra in patients with acute pericarditis. J Cardiovasc Pharmacol. (2020) 76:50–2. doi: 10.1097/FJC.0000000000000847 32398478

[197] Healy AH Bowen M McKellar SH Koliopoulou A Drakos SG Selzman CH. Interleukin-1 receptor antagonism as adjunct therapy for heart failure patients with left ventricular assist devices. ASAIO J (American Soc Artif Internal Organs. (2021) 1992) 67:e145–7. doi: 10.1097/MAT.0000000000001347 33470637

[198] Van Tassell BW Trankle CR Canada JM Carbone S Buckley L Kadariya D. IL-1 blockade in patients with heart failure with preserved ejection fraction. Circulation Heart Failure. (2018) 11:e005036. doi: 10.1161/CIRCHEARTFAILURE.118.005036 30354558 PMC6545106

[199] Abbate A Van Tassell BW Biondi-Zoccai G Kontos MC Grizzard JD Spillman DW. Effects of interleukin-1 blockade with anakinra on adverse cardiac remodeling and heart failure after acute myocardial infarction [from the Virginia Commonwealth University-Anakinra Remodeling Trial (2) (VCU-ART2) pilot study. Am J Cardiol. (2013) 111:1394–400. doi: 10.1016/j.amjcard.2013.01.287 PMC3644511 23453459

[200] Van Tassell BW Lipinski MJ Appleton D Roberts CS Kontos MC Abouzaki N. Rationale and design of the Virginia Commonwealth University-Anakinra Remodeling Trial-3 (VCU-ART3): A randomized, placebo-controlled, double-blinded, multicenter study. Clin Cardiol. (2018) 41:1004–8. doi: 10.1002/clc.2018.41.issue-8 PMC6153042 30033595

[201] Nidorf SM Eikelboom JW Budgeon CA Thompson PL. Low-dose colchicine for secondary prevention of cardiovascular disease. J Am Coll Cardiol. (2013) 61:404–10. doi: 10.1016/j.jacc.2012.10.027 23265346

[202] Nidorf SM Fiolet ATL Mosterd A Eikelboom JW Schut A Opstal TSJ. Colchicine in patients with chronic coronary disease. New Engl J Med. (2020) 383:1838–47. doi: 10.1056/NEJMoa2021372 32865380

[203] Tardif JC Kouz S Waters DD Bertrand OF Diaz R Maggioni AP. Efficacy and safety of low-dose colchicine after myocardial infarction. New Engl J Med. (2019) 381:2497–505. doi: 10.1056/NEJMoa1912388 31733140

[204] Mewton N Roubille F Bresson D Prieur C Bouleti C Bochaton T. Effect of colchicine on myocardial injury in acute myocardial infarction. Circulation. (2021) 144:859–69. doi: 10.1161/CIRCULATIONAHA.121.056177 PMC8462445 34420373

[205] Ridker PM Everett BM Thuren T MacFadyen JG Chang WH Ballantyne C. Antiinflammatory therapy with canakinumab for atherosclerotic disease. New Engl J Med. (2017) 377:1119–31. doi: 10.1056/NEJMoa1707914 28845751

[206] Ridker PM MacFadyen JG Thuren T Everett BM Libby P Glynn RJ. Effect of interleukin-1β inhibition with canakinumab on incident lung cancer in patients with atherosclerosis: exploratory results from a randomised, double-blind, placebo-controlled trial. Lancet (London England). (2017) 390:1833–42. doi: 10.1016/S0140-6736(17)32247-X 28855077

[207] Napoli C Hayashi T Cacciatore F Casamassimi A Casini C Al-Omran M. Endothelial progenitor cells as therapeutic agents in the microcirculation: an update. Atherosclerosis. (2011) 215:9–22. doi: 10.1016/j.atherosclerosis.2010.10.039 21126740

[208] Klein AL Imazio M Brucato A Cremer P LeWinter M Abbate A. RHAPSODY: Rationale for and design of a pivotal Phase 3 trial to assess efficacy and safety of rilonacept, an interleukin-1α and interleukin-1β trap, in patients with recurrent pericarditis. Am Heart J. (2020) 228:81–90. doi: 10.1016/j.ahj.2020.07.004 32866928

